# Molecular basis underlying the specificity of an antagonist AA92593 for mammalian melanopsins

**DOI:** 10.1016/j.jbc.2025.108461

**Published:** 2025-03-26

**Authors:** Kohei Obayashi, Ruisi Zou, Tomoki Kawaguchi, Toshifumi Mori, Hisao Tsukamoto

**Affiliations:** 1 Department of Biology, Graduate School of Science, Kobe University, Kobe, Japan; 2 Interdisciplinary Graduate School of Engineering Sciences, Kyushu University, Fukuoka, Japan; 3 Institute for Materials Chemistry and Engineering, Kyushu University, Fukuoka, Japan; 4 Center of Optical Scattering Image Science, Kobe University, Kobe, Japan

**Keywords:** melanopsin, opsin, GPCR, antagonist, molecular dynamics, non-visual photoreception

## Abstract

Melanopsin functions in intrinsically photosensitive retinal ganglion cells of mammals to regulate circadian clock and pupil constriction. The opsinamide AA92593 has been reported to specifically inhibit mouse and human melanopsin functions as a competitive antagonist against retinal; however, the molecular mechanisms underlying its specificity have not been resolved. In this study, we attempted to identify amino acid residues responsible for the susceptibility of mammalian melanopsins to AA92593. Our cell-based assays confirmed that AA92593 effectively inhibited the light-induced cellular responses of mammalian melanopsins, but not those of non-mammalian vertebrate and invertebrate melanopsins. These results suggest that amino acid residues specifically conserved among mammalian melanopsins are important for the antagonistic effect of AA92593, and we noticed Phe-94^2.61^, Ser-188^ECL2^, and Ser-269^6.52^ as candidate residues. Substitutions of these residues reduced the antagonistic effect of AA92593. We conducted docking and molecular dynamics simulations based on the AlphaFold-predicted melanopsin structure. The simulations indicated that Phe-94^2.61^, Ser-188^ECL2^, and Ser-269^6.52^ are located at the AA92593-binding site and additionally identified Trp-189^ECL2^ and Leu-207^5.42^ interacting with the antagonist. Substitutions of Trp-189^ECL2^ and Leu-207^5.42^ affected the antagonistic effect of AA92593. Furthermore, substitutions of these amino acid residues converted the AA92593-insensitive non-mammalian melanopsins susceptible to the antagonist. Based on experiments and molecular simulations, five amino acid residues, at positions 94^2.61^, 188^ECL2^, 189^ECL2^, 207^5.42^, and 269^6.52^, were found to be responsible for the specific susceptibility of mammalian melanopsins to AA92593.

In mammals, melanopsin (or Opn4) is expressed in intrinsically photosensitive retinal ganglion cells (ipRGCs) and plays important roles in non-visual photoreceptive functions such as circadian photoentrainment and pupil constriction (1, 2). Melanopsin is a blue light-sensitive G protein-coupled receptor (GPCR) and a member of the opsin family (3, 4). Light-induced regulation of melanopsin function has been utilized in neuroscience, behavioral, and clinical studies (5, 6, 7). Melanopsin functions can also be manipulated by chemicals. An opsinamide AA92593 (see Fig. 1*B*) was reported to be a competitive antagonist against the chromophore retinal, specifically acting on melanopsin (8). The original study clearly showed that AA92593 effectively antagonized the activity of melanopsin-expressing ipRGCs, but not visual photoreceptor cells. Since then, several studies have used AA92593 to suppress melanopsin function chemically (5, 9, 10).

**Figure 1:**
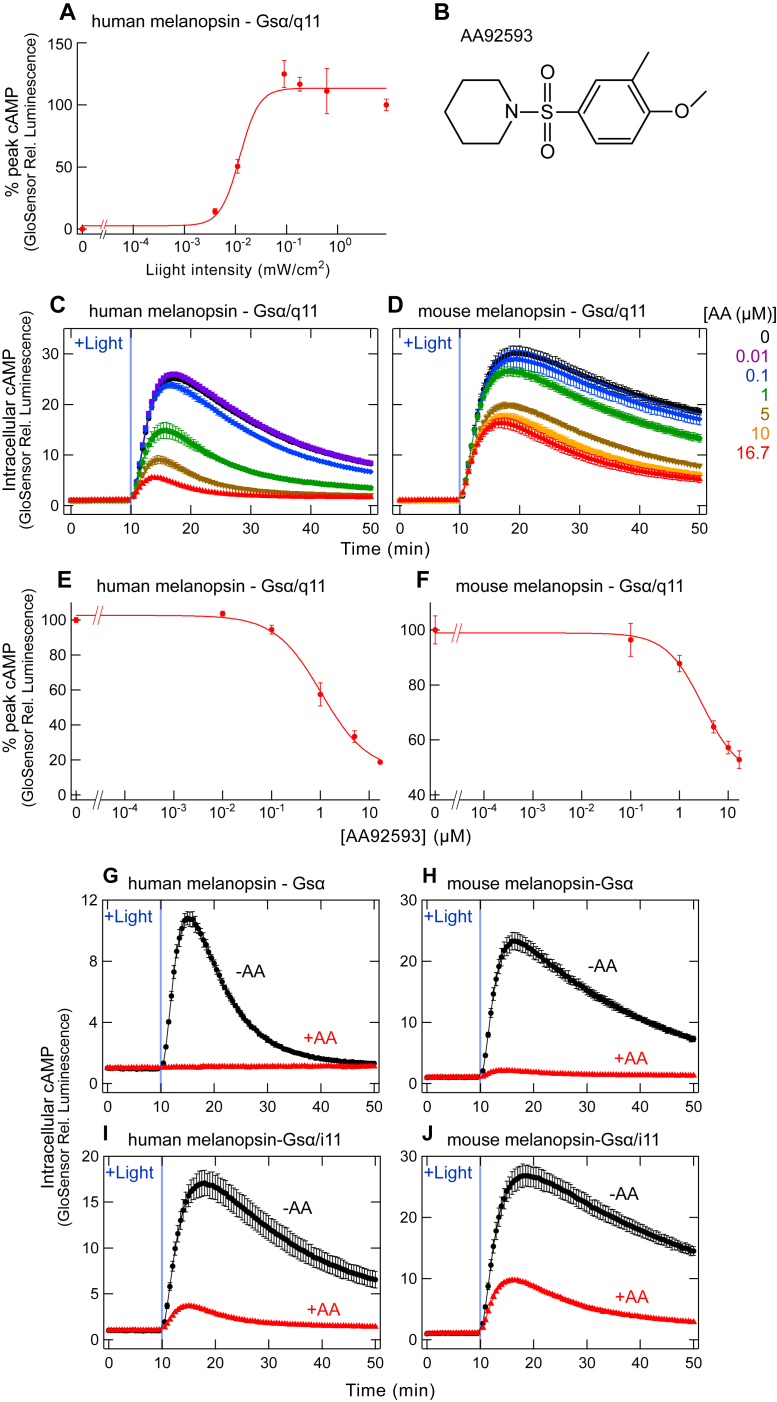
**AA92593-induced suppression of G protein activation by mammalian melanopsins assessed by GsX GloSensor assay.***A*, light stimulus–response curves of GsX GloSensor assay for human melanopsin with Gsα/q11. Error bars indicate the SD values (n = 3). Fitting parameters: *max*, 113.43 ± 5.86; *min*, 2.71 ± 11.1; *rate*, 2.30 ± 1.85. The EC_50_ value of the curve is ∼10 μW/cm^2^. *B*, chemical structure of AA92593 (8). *C* and *D*, AA92593 concentration-dependent inhibition of intracellular cAMP elevation in COS-1 cells upon Gsα/q11 activation of human (*C*) and mouse (*D*) melanopsins. Luminescence levels of cAMP biosensor (GloSensor) are normalized to the values at the starting point (time = 0 min). In (*C*) and (*D*), final AA92593 concentrations are 16.7 (*red*), 10 (*orange*), 5 (*brown*), 1 (*green*), 0.1 (*blue*), 0.01 (*purple*), and 0 μM (*black*), respectively. *Light blue* bars indicate *white* light illumination (10 s). Error bars indicate the SD values (n = 3). *E* and *F*, dose-dependent reduction in peak cAMP responses upon Gsα/q11 activation in human (*E*) and mouse (*F*) melanopsin-expressing COS-1 cells. Relative average peak values to the value in the absence of AA92593 are plotted against the final concentrations of AA92593. Error bars indicate the SD values (n = 3). Fitting parameters for human melanopsin: *max*, 102.59 ± 2.72; *min*, 13.65 ± 7.28; *rate*, −0.92 ± 0.21. Fitting parameters for mouse melanopsin: *max*, 98.86 ± 1.11; *min*, 46.53 ± 4.15; *rate*, −1.15 ± 0.18. IC_50_ values for human and mouse melanopsins are 1.05 ± 0.28 μM and 2.98 ± 0.58 μM, respectively. *G* and *H*, AA92593-dependent inhibition of intracellular cAMP elevation in COS-1 cells upon Gsα activation of human (*G*) and mouse (*H*) melanopsins. Error bars indicate the SD values (n = 3). *I* and *J*, AA92593-dependent inhibition of intracellular cAMP elevation in COS-1 cells upon Gsα/i11 activation of human (*I*) and mouse (*J*) melanopsins. Error bars indicate the SD values (n = 3). In (*G*–*J*), *red* and *black* curves indicate luminescence changes in the presence and absence of 16.7 μM AA92593, respectively. *Light blue* bars indicate *white* light illumination (10 s). Luminescence levels of cAMP biosensor (GloSensor) are normalized to the values at the starting point (time = 0 min).

Although effectiveness of AA92593 as a melanopsin antagonist has been proven, it is unclear how AA92593 selectively blocks the photoresponse of mammalian-type melanopsin (Opn4m) and whether the antagonist is effective for closely related opsins such as non-mammalian-type melanopsin (Opn4x). To understand the specificity of AA92593 for mammalian melanopsins, it is important to assess the antagonistic effect on various melanopsins under the same experimental conditions and to identify the amino acid residues responsible for the specificity *via* site-directed mutagenesis. Unfortunately, despite the recent progress in GPCR structural biology (11), structural information about melanopsin is still limited. In this regard, sequence alignment of melanopsins and other opsins, structure prediction, and molecular simulations can be utilized to reveal the molecular interactions from structural perspectives.

In the present study, we aimed to reveal the effectiveness of AA92593 as an antagonist for a wide variety of melanopsins and the molecular mechanisms underlying the effectiveness. In particular, we identified the amino acid residues in melanopsins responsible for the specific antagonistic effect of AA92593. To do so, we performed several cell-based assays on human and mouse melanopsins as well as closely-related opsins to evaluate their susceptibility for the antagonist. We also conducted the docking and molecular dynamics (MD) simulations of human melanopsin with AA92593, based on an AlphaFold-predicted structure, to visualize the binding mode of the antagonist in the melanopsin. Our experiments indicated that AA92593 is specifically effective on mammalian melanopsins but not on other opsins. Our mutational experiments and computational simulations consistently indicated that five amino acid residues near the retinal-binding site in mammalian melanopsin directly interact with AA92593 to regulate its effectiveness. Substitutions of the corresponding amino acid residues in AA92593-insensitive non-mammalian and invertebrate melanopsins increased their susceptibility to the antagonist. Our data revealed how AA92593 specifically acts as an antagonist against mammalian melanopsins, and the data provide valuable information for engineering melanopsins to be regulated effectively (or ineffectively) by the antagonist.

## Results

### Assessment of antagonistic effects of AA92593 on mammalian melanopsin (Opn4m) using GsX GloSensor assays

To quantify the antagonistic effects of AA92593 on mammalian melanopsins, we used the GsX GloSensor assay (12, 13, 14). This assay measures GPCR-induced increases in intracellular cAMP levels even for Gq-, Gi/o-, or G12-coupled receptors by using Gsα chimeras (12, 13, 14). Because melanopsin is primarily coupled with Gq-type G proteins, a Gsα mutant with 11 C-terminal sequence of Gqα (Gsα/q11) was expressed in COS-1 cells with melanopsin and a luciferase-based cAMP biosensor (see “Experimental procedures”) (15). This assay enables the measurement of Gq activation by melanopsin as cAMP production (an increase in luminescence). In this study, we used C-terminal–truncated melanopsin constructs to increase the expression levels with minimal compromise in functionality (9, 16, 17, 18, 19). Previous studies of melanopsin have reported that the C-terminal truncation does not affect the photoreaction or G protein activation of melanopsins. In addition, the C-terminal region is located far from the binding site of AA92593 (see below). Hereafter, the C-terminal–truncated melanopsin constructs are named as “WT.”

We conducted the GsX GloSensor assay using Gsα/q11 upon various intensity of light to activate human melanopsin WT (Fig. 1*A*). Our GsX GloSensor assay detected light-dependent Gsα/q11 activation by human and mouse melanopsins (see Fig. 1, *C* and *D*), and the results indicated that the EC_50_ value of light intensity was ∼10 μW/cm^2^. For other experiments (Fig. 1*A*), we used 9 mW/cm^2^ intensity (∼1000-fold of the EC_50_ value) of light to compare saturated cellular responses by melanopsins.

Our GsX GloSensor assay detected the inhibition of human and mouse melanopsins by AA92593 in a concentration-dependent manner (Fig. 1, *B*–*D*). The IC_50_ values for human and mouse melanopsins were 1.05 ± 0.28 μM and 2.98 ± 0.58 μM, respectively (Fig. 1, *E* and *F*). The IC_50_ value for human melanopsin in our experiments is somewhat different from the reported value in the previous study (665 nM for human melanopsin) (8). The differences are probably due to experimental conditions and methods such as cell lines (CHO *versus* COS-1), cellular assays detecting melanopsin activity (calcium imaging *versus* GsX GloSensor assay), and/or incubation time with 9-*cis*-retinal (several minutes *versus* about 1 hour).

Previous studies of human and mouse melanopsins reported that these melanopsins can activate not only Gq but Gs and Gi (20, 21, 22). Thus, we assessed the effect of AA92593 on Gsα WT or Gsα/i11 (with 11 C-terminal sequence of Giα) using the GsX GloSensor assay. In agreement with the promiscuous G protein-coupling of melanopsins, we observed light-dependent increase of GloSensor luminescence in COS-1 cells expressing Gsα or Gsα/i11 with human/mouse melanopsins (Fig. 1, *G*–*J*). The results also indicated that AA92593 can suppress melanopsin-induced cAMP responses *via* exogenous Gsα (Fig. 1, *G* and *H*) or Gsα/i11 (Fig. 1, *I* and *J*). In addition, human melanopsin induced light-dependent cAMP responses *via* endogenous G proteins in COS-1 cells, and the endogenous G protein-induced cAMP responses were also inhibited by the addition of AA92593. The endogenous G proteins produced much less GloSensor luminescence signals, probably due to the low expression levels of endogenous G proteins (Fig. S1), suggesting that the GloSensor signals were predominantly caused by transfected G protein constructs rather than endogenous G proteins. These results showed that the activation of Gq, Gs, and Gi by mammalian melanopsins was suppressed by AA92593, which is consistent with the previous study showing that the molecule suppresses melanopsin signaling by the competitive binding against retinal (8) but not by blocking of G protein binding.

### Conserved amino acid residues important for susceptibility to AA92593

**Figure 2:**
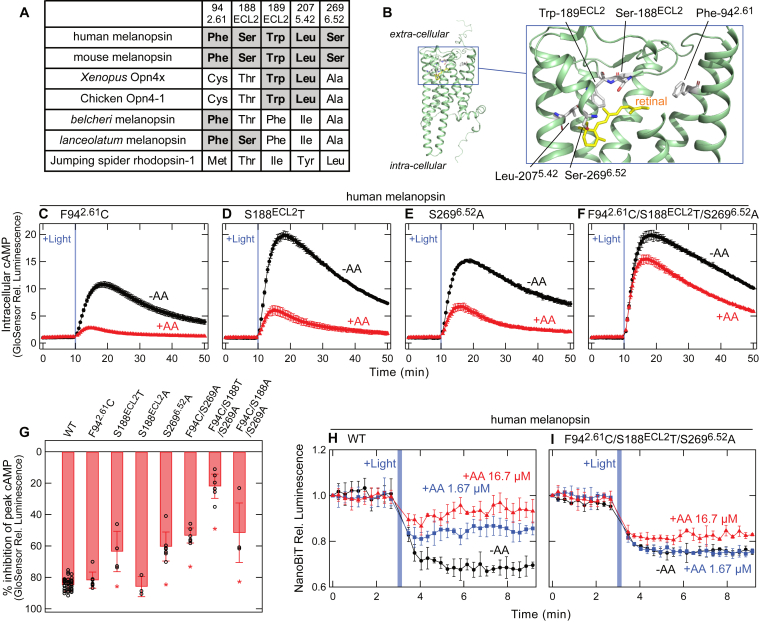
**Changes in the susceptibility of human melanopsin for AA92593 by substitutions of conserved amino acid residues in the putative retinal-binding site.***A*, amino acid residues at positions 94^2.61^, 188^ECL2^, 189^ECL2^, 207^5.42^, and 269^6.52^ in various melanopsins and a non-melanopsin Gq-coupled opsin. “Mammalian melanopsin-type” amino acids are highlighted as bold. The amino acid residue numbering in this paper is based on the amino acid sequence of bovine rhodopsin. *B*, arrangement of the amino acid residues at positions 94^2.61^, 188^ECL2^, 189^ECL2^, 207^5.42^, and 269^6.52^ in the AlphaFold2-predicted human melanopsin structure (58). The 11-*cis*-retinal molecule is adopted from the crystal structure of bovine rhodopsin (PDB ID: 1U19) (59). Note that the predicted structure of human melanopsin and the crystal structure of bovine rhodopsin with 11-*cis*-retinal were overlapped to obtain a structure that looks like retinal bound to human melanopsin. The structural models are prepared using PyMOL (https://pymol.org/). *C*-*F*, AA92593-dependent inhibition of intracellular cAMP elevation in COS-1 cells upon Gsα/q11 activation of human melanopsin mutants F94^2.61^C (*C*), S188^ECL2^T (*D*), S269^6.52^A (*E*), and F94^2.61^C/S188^ECL2^T/S269^6.52^A (*F*). *Red* and *black* traces indicate luminescence changes in the presence and absence of 16.7 μM AA92593, respectively. *Light blue* bars indicate *white* light illumination (10 s). Luminescence levels of cAMP biosensor (GloSensor) are normalized to the values at the starting point (time = 0 min). Error bars indicate the SD values (n = 3). *G*, comparison of inhibition in peak cAMP responses by 16.7 μM AA92593 upon Gsα/q11 activation in human melanopsin WT and mutants. Error bars indicate the SD values of independent experiments (n = 46, 8, 4, 3, 7, 6, 7, and 4 for WT, F94^2.61^C, S188^ECL2^T, S188^ECL2^A, S269^6.52^A, F94^2.61^C/S269^6.52^A, F94^2.61^C/S188^ECL2^T/S269^6.52^A, and F94^2.61^C/S188^ECL2^A/S269^6.52^A, respectively). The statistical *p* values in differences from WT are 0.99, <0.00001∗, 1.00, <0.00001∗, <0.00001∗, <0.00001∗, and <0.00001∗ for F94^2.61^C, S188^ECL2^T, S188^ECL2^A, S269^6.52^A, F94 C/S269^6.52^A, F94^2.61^C/S188^ECL2^T/S269^6.52^A, and F94^2.61^C/S188^ECL2^A/S269^6.52^A, respectively (Dunnett's test following one-way ANOVA, F = 79, d. f. = 14, 91). *H* and *I*, NanoBiT Gq dissociation assay using Gqα/R183Q-LgBiT on human melanopsin WT (*H*) and F94^2.61^C/S188^ECL2^T/S269^6.52^A mutant (*I*). *Red*, *blue*, and *black* traces indicate luminescence changes in the presence of 16.7 μM (*red*), 1.67 μM (*blue*), and 0 μM (*black*) AA92593. *Light blue* bars indicate *white* light illumination (10 s). NanoLuc luminescence levels are normalized to the values at the starting point (time = 0 min). Error bars indicate the SD values (n = 3).

Because the inhibitory effect of AA92593 was detected by the GsX GloSensor assay, we attempted to identify the amino acid residues important for the susceptibility to AA92593. Because AA92593 competes with the retinal chromophore for binding to mammalian melanopsins (8), we focused on the amino acid residues near the retinal-binding site that are specifically conserved among mammalian melanopsins. Based on sequence alignment of amino acid residues constituting the putative retinal-binding site (Fig. S2), three residues, Phe-94^2.61^, Ser-188^ECL2^, and Ser-269^6.52^, were found to be conserved in mammalian-type melanopsin (Opn4m) (Figs. 2*A* and S2). Note that hereafter, we described the amino acid residue numbers using bovine rhodopsin numbering with Ballesteros/Weinstein (GPCRdb) numbering as superscript (Fig. S3). In non-mammalian-type melanopsin (Opn4x), these positions are not conserved and are occupied by Cys, Thr, and Ala, respectively (Figs. 2*A*, S2 and S3).

**Figure 3:**
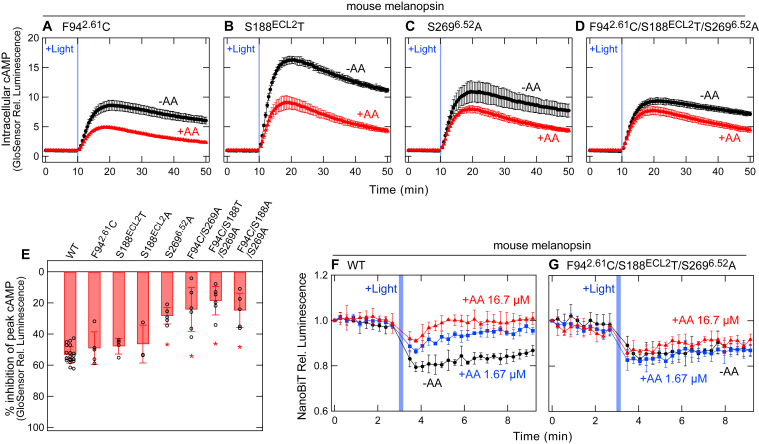
**Changes in the susceptibility of mouse melanopsin for AA92593 by substitutions in the putative retinal-binding site.***A*–*D*, AA92593-dependent inhibition of intracellular cAMP elevation in COS-1 cells upon Gsα/q11 activation of mouse melanopsin mutants F94^2.61^C (*A*), S188^ECL2^T (*B*), S269^6.52^A (*C*), and F94^2.61^C/S188^ECL2^T/S269^6.52^A (*D*). *Red* and *black* traces indicate luminescence changes in the presence and absence of 16.7 μM AA92593, respectively. *Light blue* bars indicate white light illumination (10 s). Luminescence levels of cAMP biosensor (GloSensor) are normalized to the values at the starting point (time = 0 min). Error bars indicate the SD values (n = 3). *E*, comparison of inhibition in peak cAMP responses by 16.7 μM AA92593 upon Gsα/q11 activation in mouse melanopsin WT and mutants. Error bars indicate the SD values of independent experiments (n = 23, 5, 4, 3, 5, 6, 6, and 5 for WT, F94^2.61^C, S188^ECL2^T, S188^ECL2^A, S269^6.52^A, F94^2.61^C/S269^6.52^A, F94^2.61^C/S188^ECL2^T/S269^6.52^A and F94^2.61^C/S188^ECL2^A/S269^6.52^A, respectively). The statistical *p* values in differences from WT are 0.95, 0.88, 0.81, <0.00001∗, <0.00001∗, <0.00001∗, and <0.00001∗ for F94^2.61^C, S188^ECL2^T, S188^ECL2^A, S269^6.52^A, F94^2.61^C/S269^6.52^A, F94^2.61^C/S188^ECL2^T/S269^6.52^A, and F94^2.61^C/S188^ECL2^A/S269^6.52^A, respectively (Dunnett's test following one-way ANOVA, F = 33, d. f. = 9, 53). *F* and *G*, NanoBiT Gq dissociation assay on mouse melanopsin WT (*F*) and F94^2.61^C/S188^ECL2^T/S269^6.52^A mutant (*G*). *Red*, *blue*, and *black* traces indicate luminescence changes in the presence of 16.7 μM (*red*), 1.67 μM (*blue*), and 0 μM (*black*) AA92593. *Light blue* bars indicate *white* light illumination (10 s). NanoLuc luminescence levels are normalized to the values at the starting point (time = 0 min). Error bars indicate the SD values (n = 3).

We substituted the residues Phe-94^2.61^, Ser-188^ECL2^, and Ser-269^6.52^ in human melanopsin (Fig. 2, *A* and *B*) and examined the inhibitory effect of AA92593 on these mutants. Substitution of these amino acid residues with the corresponding amino acids in non-mammalian-type melanopsins (F94^2.61^C, S188^ECL2^T, and S269^6.52^A) reduced the inhibitory effect of AA92593 compared to its effect on WT (Fig. 2, *C*–*E*). While statistically significant difference was not observed between the F94^2.61^C mutant and WT, the inhibitory effect was consistently weaker for the mutant in each experiment (Figs. 2, *C* and *G* and S4). The antagonistic effect of AA92593 was reduced by ∼21% and ∼24% in the S188^ECL2^T and S269^6.52^A mutants, respectively (Fig. 2, *D,* *E*, and *G*). Unlike the S188^ECL2^T substitution, the S188^ECL2^A substitution did not induce a significant change in the effect of AA92593 (Fig. 2*G*), suggesting that the size of the Ser residue is important for the antagonistic effect rather than the hydroxyl group of the side chain. The volume change in side chain would affect arrangement of the amino acid residues around AA92593 (see below). Similar results were observed for the mouse melanopsin F94^2.61^C, S188^ECL2^T, and S269^6.52^A mutants (Fig. 3, *A*–*C*, and *E*), although only the S269^6.52^A mutant showed a statistically significant difference (∼25% reduction) from WT (see below).

The single substitutions F94^2.61^C, S188^ECL2^T, and S269^6.52^A in mammalian melanopsins reduced the inhibitory effects of AA92593 (Figs. 2*G* and 3*E*). We suspected that the combination of these substitutions would further decrease the AA92593 effect. We thus introduced the triple substitutions F94^2.61^C/S188^ECL2^T/S269^6.52^A into human and mouse melanopsins and examined the inhibitory effects of AA92593. In both mammalian melanopsins, the F94^2.61^C/S188^ECL2^T/S269^6.52^A substitution reduced the inhibitory effect of AA92593 more than the single substitutions (∼63% and ∼35% reductions for human and mouse melanopsins, respectively) (Figs. 2, *F* and *G*, 3, *D* and *E*). These results clearly indicated that the effects of Phe-94^2.61^, Ser-188^ECL2^, and Ser-269^6.52^ on the susceptibility to AA92593 are additive in the mammalian melanopsins.

As described above, we have assessed the inhibitory effect of AA92593 using the GsX GloSensor assay. To confirm whether an antagonistic effect could be observed in another experimental system, we used the NanoBiT G protein dissociation assay (23). In this assay, Gq activation (dissociation) is detected as a decrease in NanoLuc luminescence from the Lg-BiT fragment inserted with Gqα and the Sm-BiT fragment fused with Gβγ (see “Experimental procedures”) (23, 24). If an antagonistic effect is observed in the NanoBiT assay, the light-dependent decrease in luminescence (Gq dissociation) by melanopsin would be reduced in the presence of AA92593. As expected, human melanopsin WT showed a light-dependent decrease in luminescence intensity (Fig. S5), indicating that Gq activation by the melanopsin was successively detected using the NanoBiT assay. A recent study reported that the introduction of a GTPase-deficient substitution, R183Q, on Gqα increased GPCR-induced Gq dissociation signals in a BRET-based biosensor assay, TRUPATH (25). Based on this insight, we prepared the Gqα with Lg-BiT containing the R183Q mutation and confirmed that melanopsin-induced Gq dissociation signals on NanoBiT assay was larger than Gqα WT with Lg-BiT (Fig. S5), probably because of the suppression of Gq inactivation in the Gqα R183Q mutant. We used the Gqα/R183Q-LgBiT to compare the signals induced by melanopsin mutants, with or without AA92593.

The light-dependent decrease in luminescence was weakened by the addition of AA92593 in a concentration-dependent manner (Fig. 2*H*). The human melanopsin mutant F94^2.61^C/S188^ECL2^T/S269^6.52^A also showed a robust light-dependent decrease in NanoLuc luminescence intensity. However, the AA92593-dependent inhibitory effect on Gq activation by the mutant was much reduced compared to that on WT (Fig. 2*I*). The antagonistic effect on Gq activation by mouse melanopsin was similarly reduced by the F94^2.61^C/S188^ECL2^T/S269^6.52^A substitutions (Fig. 3, *F* and *G*). These NanoBiT assay data clearly indicate that the antagonistic effect of AA92593 on mammalian melanopsins was observed at the Gq activation levels, which supports our conclusion that Phe-94^2.61^, Ser-188^ECL2^, and Ser-269^6.52^ play important roles in the susceptibility.

### Docking and MD simulations of human melanopsin with AA92593

**Figure 4:**
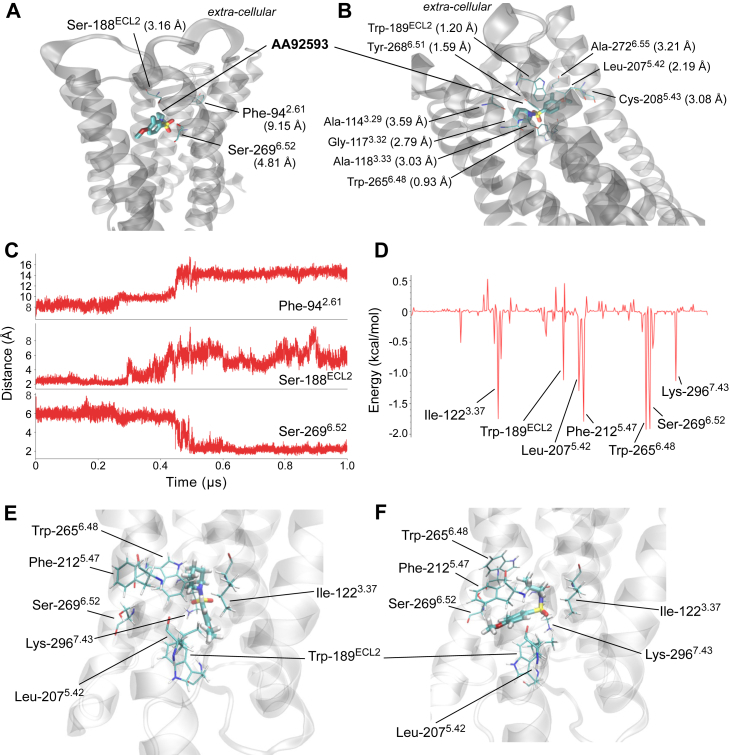
**Docking and MD simulations on human melanopsin with AA92593.***A* and *B*, structure of the human melanopsin–AA92593 complex predicted from DiffDock. Parenthesis show the shortest distances between AA92593 and selected ligands. The distances in (*A*) and (*B*) are between heavy atoms, whereas those in other panels are between all atoms including hydrogen. *C*, time evolution of the distances between AA92593 and Phe-94^2.61^, Ser-188^ECL2^, and Ser-269^6.52^ over the 1 μs MD trajectory. *D*, per-residue decomposition of the binding energy calculated using MMPBSA. *E* and *F*, structure of the melanopsin–AA92593 complex before (*E*) and after (*F*) the 1 μs MD simulation.

To visualize how Phe-94^2.61^, Ser-188^ECL2^, and Ser-269^6.52^ make mammalian melanopsin susceptible to AA92593, we conducted computational studies on the human melanopsin–AA92593 complex. Despite recent progress in the structural analyses of various GPCRs, the structure of melanopsin has not yet been experimentally solved. Thus, we adopted the AlphaFold-predicted structural model of human melanopsin and performed the docking analysis with AA92593. Calculations using AutoDock (26) were unsuccessful in obtaining the ligand bound to the pocket of human melanopsin, possibly because of steric clashes with the side-chains (27) (Fig. S6). In contrast, by performing the blind docking calculation using DiffDock (28), we obtained the structure of the complex in which the ligand was located in the retinal-binding pocket of the human melanopsin (Fig. 4, *A* and *B*). The receptor-ligand docked structure with the best score clearly indicated that Ser-188^ECL2^ and Ser-269^6.52^ are near the binding site of AA92593, where the piperidine and benzene rings of AA92593 (see Fig. 1*B*) were ∼3.2 and ∼4.8 Å from Ser-188^ECL2^ and Ser-269^6.52^, respectively (Fig. 4*A*). On the other hand, Phe-94^2.61^ is away from AA92593 by ∼9.2 Å. Note that these distances in the docked structures are measured between the heavy atoms due to the lack of hydrogens in the structure. (Fig. 4*A*). These locations were consistent with our experiments, showing that single substitutions of S188^ECL2^T and S269^6.52^A significantly reduced the antagonistic effect, whereas the single F94C substitution resulted in a minor reduction (Fig. 2*G*). In addition, Ala-114^3.29^, Gly-117^3.32^, Ala-118^3.33^, Trp-189^ECL2^, Leu-207^5.42^, Cys-208^5.43^, Trp-265^6.48^, Tyr-268^6.51^, and Ala-272^6.55^ were found to be located within 4 Å from the ligand (Fig. 4*B*). In particular, the benzene ring and the two oxygen atoms in the SO_2_ group of AA92593 were within a van der Waals radius of the indole ring of Trp-189 ^ECL2^, indole ring of Trp-265^6.48^, and phenyl ring of Tyr-268^6.51^, respectively. On the other hand, the piperidine ring moiety was loosely packed, implying that it could rotate easily. We also note that no hydrogen bond was found in the complex structure. The close contacts, for example, between Trp-265^6.48^ and AA92593, indicate that some structural rearrangements are likely to occur when the structure of the complex is relaxed.

Since the ligand in the binding pocket predicted in the crystal structures may not always be rigid at the binding pocket under the physiological conditions (29, 30), and the protein environment of the ligand may also show conformational changes over time (31, 32), we next explored the dynamics of the melanopsin–AA92593 interactions in the complex by performing MD simulations (Fig. 4, *C*–*F*, S7 and S8). The initial structure was obtained from the docking model, and the membrane and solvents were added as described below. Due to the close distance between Trp-265^6.48^ and AA92593, the ligand was found away from Trp-265^6.48^ and Ser-269^6.52^ at the end of equilibration (Fig. 4, *C* and *E*). Yet, after 448 ns, the ligand moved back into the pocket between Trp-189^ECL2^ and Trp-265^6.48^ and stayed within ∼3 Å from Ser-269^6.52^ while relaxing to the optimal binding position (Figs. 4, *C* and *F*, and S8). The ligand was initially near Ser-188^ECL2^ (∼3 Å) but gradually moved away after 0.3 μs (Fig. 4, *C* and *F*). Phe-94^2.61^ was >7 Å away from the ligand (Fig. 4*C*). These trends are consistent with the docking model. The per-residue decomposition of the binding energy, obtained by the molecular mechanics Poisson-Boltzmann surface area (MMPBSA) calculations for the last 300 ns of the trajectory (which we considered as the relaxed binding position), indicated that Ile-122^3.37^, Trp-189^ECL2^, Leu-207^5.42^, Phe-212^5.47^, Trp-265^6.48^, Ser-269^6.52^, and Lys-296^7.43^ markedly contribute to stabilizing binding (Fig. 4*D*). Van der Waals interactions was found to be predominant, except for Ser-269^6.52^ and Lys-296^7.43^ in which electrostatic interaction showed notable contribution (Fig. S7). The distances between the ligand and these residues were mostly within 3 Å after the ligand moved into the optimal position (Figs. 4*F* and S8). Notably, not all residues in contact with the ligand showed a large binding energy (*e.g.*, Cys-208^5.43^) (Fig. 4*B*), implying that both the distance and character of the residues were important for stabilization.

We further inspected the molecular interactions behind the MMPBSA per-residue decomposition energies. The origin of the electrostatic interaction for Ser-269^6.52^ was found to be the hydrogen bond between the oxygen in the methoxy group in AA92593 and hydroxyl group of Ser-269^6.52^, which was formed in ∼40% of the trajectory in the optimal binding position (from 450 ns to 1 μs) (Fig. 4*C*). Ile-122^3.37^ stayed near the SO_2_ group of AA92593 (Fig. 4, *E* and *F*); Trp-189^ECL2^ and Leu-207^5.42^ were near the benzene ring (Fig. 4*F*), and Trp-189^ECL2^ occasionally showed weak CH-π interaction between the hydrogen in the indole ring of Trp and the benzene ring in AA92593; Phe-212^6.47^ and Trp-265^6.48^ were located near the piperidine ring (Fig. 4*F*). Overall, AA92593 was sandwiched between the Trp-189 ^ECL2^ and Trp-265^6.48^ groups, and no characteristic ligand–protein interaction seemed to persist in the binding pocket.

In addition to the direct ligand–protein interactions, we also explored the hydrogen bond networks mediated by water molecules. Water molecules were not found near the ligand right after equilibration but gradually reached about the ligand as the ligand rearranged its structure during the 1 μs trajectory. After the ligand relaxed to its optimal binding position, water molecules frequently hydrogen bonded to the O2 atom of the SO_2_ group in AA92593 (Fig. 1*B*), and the hydrogen bond network connecting Ser-188^ECL2^, Tyr-268^6.51^, one to two water molecules, and O2 of AA92593 was formed (Fig. S9*A*). The radial distribution function of the hydrogens in water around the O2 atom of the ligand also suggested the presence of water-mediate hydrogen bond network about the ligand (Fig. S9*B*). Here we note that water mostly interacted with only one of the oxygen atoms in the SO_2_ group due to the heterogeneous environment about the ligand (Fig. S9*B*).

Combining the docking and MD simulations of the human melanopsin in complex with AA92593 identified several amino acid residues that presumably interact with the antagonist in addition to Phe-94^2.61^, Ser-188^ECL2^, and Ser-269^6.52^. In particular, Trp-189^ECL2^, Leu-207^5.42^, and Trp-265^6.48^, which are conserved among not only mammalian-type but also non-mammalian-type melanopsins (Figs. S2 and S3), were found to be important for AA92593 binding in both the docking and MD results (Figs. 4 and S8). Because Trp-265^6.48^ is highly conserved in GPCRs and has been reported to be important for proper function and/or folding in many GPCRs including opsins, the Trp-265 substitution is likely to severely impair receptor functions (33, 34, 35, 36). Thus, we assessed whether substitutions of Trp-189^ECL2^ and Leu-207^5.42^ affect the susceptibility of human melanopsin to AA92593.

### Experimental assessment of interaction of Trp-189^ECL2^ and Leu-207^5.42^ with AA92593

**Figure 5:**
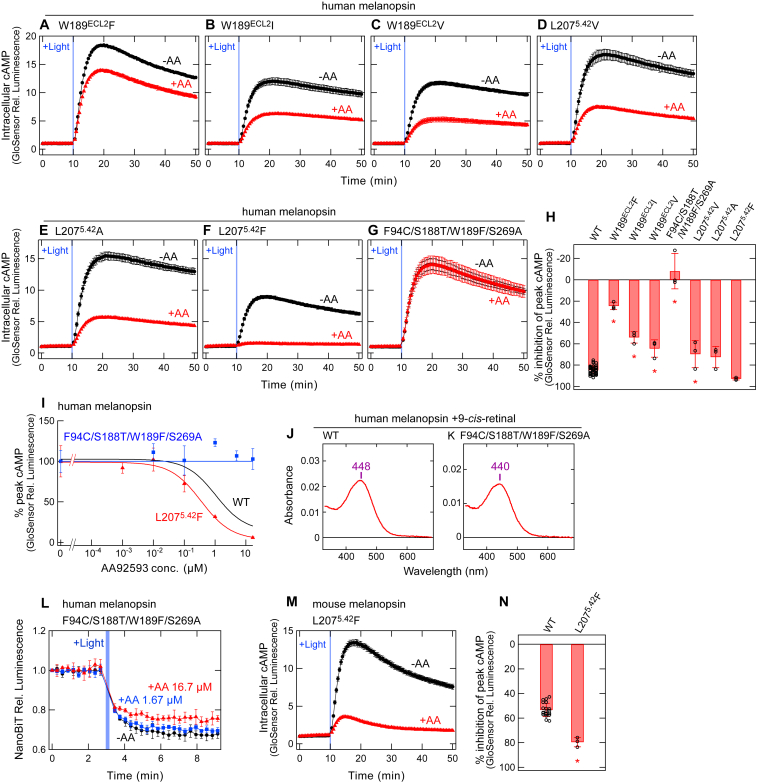
**Changes in the susceptibility of human melanopsin for AA92593 by substitutions of amino acid residues interacting with AA92593 in simulations.***A*–*G*, AA92593-dependent inhibition of intracellular cAMP elevation in COS-1 cells upon Gsα/q11 activation of human melanopsin mutants W189^ECL2^F (*A*), W189^ECL2^I (*B*), W189^ECL2^V (*C*), L207^5.42^V (*D*), L207^5.42^A (*E*), L207^5.42^F (*F*), and F94^2.61^C/S188^ECL2^T/W189^ECL2^F/S269^6.52^A (*G*). Error bars indicate the SD values (n = 3). *H*, comparison of inhibition in peak cAMP responses by 16.7 μM AA92593 upon Gsα/q11 activation in human melanopsin WT and mutants. WT data is the same as Figure 2*G*. Error bars indicate the SD values of independent experiments (n = 46, 3, 3, 3, 3, 3, 3, and 3 for WT, W189^ECL2^F, W189^ECL2^I, W189^ECL2^V, F94^2.61^C/S188^ECL2^T/W189^ECL2^F/S269^6.52^A, L207^5.42^V, L207^5.42^A, and L207^5.42^F, respectively). The statistical *p* values in differences from WT are <0.00001∗, <0.00001∗, 0.00013∗, <0.00001∗, 0.010∗, 0.072, and 0.59 for W189^ECL2^F, W189^ECL2^I, W189^ECL2^V, F94^2.61^C/S188^ECL2^T/W189^ECL2^F/S269^6.52^A, L207^5.42^V, L207^5.42^A, and L207^5.42^F, respectively (Dunnett's test following one-way ANOVA, F = 79, d. f. = 14, 91). *I*, dose-dependent reduction in peak cAMP responses upon Gsα/q11 activation by human melanopsin mutants F94^2.61^C/S188^ECL2^T/W189^ECL2^F/S269^6.52^A (*blue*) and L207^5.42^F (*red*). The curve of WT (*black*) is adopted from Figure 1*E*. Relative average peak values to the value in the absence of AA92593 are plotted against the final concentrations of AA92593. Error bars indicate the SD values (n = 3). Fitting parameters for the L207^5.42^F mutant: *max*, 98.88 ± 4.88; *min*, 3.15 ± 9.9; *rate*, −0.88 ± 0.29. IC_50_ values for the L207^5.42^F mutant is 0.35 ± 0.16 μM. *J* and *K*, absorption spectra of 9-*cis*-retinal bound human melanopsin WT (*J*) and F94^2.61^C/S188^ECL2^T/W189^ECL2^F/S269^6.52^A mutant (*K*). Respective absorption maximum (λmax) values are indicated. Our previous study reported that the 11-*cis*-retinal–bound human melanopsin WT shows the λmax at 468 nm (9). *L*, NanoBiT Gq dissociation assay using Gqα/R183Q-LgBiT on human melanopsin F94^2.61^C/S188^ECL2^T/W189^ECL2^F/S269^6.52^A mutant. *Red*, *blue*, and *black* traces indicate luminescence changes in the presence of 16.7 μM (*red*), 1.67 μM (*blue*), and 0 μM (*black*) AA92593. *Light blue* bars indicate *white* light illumination (10 s). NanoLuc luminescence levels are normalized to the values at the starting point (time = 0 min). Error bars indicate the SD values (n = 3). *M*, AA92593-dependent inhibition of intracellular cAMP elevation in COS-1 cells upon Gsα/q11 activation of mouse melanopsin L207^5.42^F mutant. Error bars indicate the SD values (n = 3). In (*A*–*G*), and (*M*), *red* and *black* traces indicate luminescence changes in the presence and absence of 16.7 μM AA92593, respectively. *Light blue* bars indicate *white* light illumination (10 s). Luminescence levels of cAMP biosensor (GloSensor) are normalized to the values at the starting point (time = 0 min). *N*, comparison of inhibition in peak cAMP responses by 16.7 μM AA92593 upon Gsα/q11 activation in mouse melanopsin WT and L207^5.42^F mutant. WT data is the same as Figure 3*E*. Error bar indicates the SD value of independent experiments (n = 23 and 3 for WT and L207^5.42^F, respectively). The statistical *p* values of L207^5.42^F in difference from WT is 0.000036∗ (Dunnett's test following one-way ANOVA, F = 33, d. f. = 9, 53).

Based on the docking and MD simulations of human melanopsin with AA92593, importance of the interactions of Trp-189^ECL2^ and Leu-207^5.42^ with the antagonist was experimentally examined using the GsX GloSensor assay. To validate the interaction between Trp-189^ECL2^ and AA92593, substitutions of W189^ECL2^F, W189^ECL2^I, or W189^ECL2^V in human melanopsin were tested. Our docking and MD simulations indicated that Trp-189^ECL2^ interact with AA92593 primarily *via* van der Waals contacts (Figs. 4*D*, S7 and S8), and we tested the effect of volume changes in the side chain *via* site-directed mutagenesis. The inhibitory effect of AA92593 was significantly weaker in all the three Trp-189^ECL2^ mutants than in WT (Fig. 5, A and B, C, and H). In particular, the W189^ECL2^F substitution resulted in the largest (∼60%) decrease in sensitivity to AA92593 compared to the other single substitutions (Fig. 5*A*). To assess the importance of Leu-207^5.42^ in the interaction with AA92593, substitutions L207^5.42^V, L207^5.42^A, or L207^5.42^F in human melanopsin were tested. The L207^5.42^V and L207^5.42^A mutants showed ∼15% and ∼12% reduced antagonistic effects of AA92593, respectively (Fig. 5, D and E, and H). Of note, the L207^5.42^F substitution enhanced the sensitivity (∼8%) of human melanopsin to AA92593 (Fig. 5*F*), although the difference was not statistically significant (Fig. 5*H*). Dose-dependent curve of reduction in the GsX GloSensor signals of the L207^5.42^F was shifted by ∼0.6 μM from the curve of WT (Fig. 5*I*). The introduction of a Phe residue at position 207^5.42^ caused additional interactions between the phenyl ring in Phe-207^5.42^ and some functional groups in AA92593. The marked changes in the antagonistic effects of Trp-189^ECL2^ and Leu-207^5.42^ substitutions are consistent with our molecular simulations, showing that these residues are located at the antagonist-binding site (Fig. 4*B*).

As mentioned above, the W189^ECL2^F mutant showed the lowest sensitivity to AA92593 among the analyzed single mutants (Fig. 5, *A* and *H*). Next, we attempted to render human melanopsin more ineffective to AA92593 by combining W189^ECL2^F with F94^2.61^C/S188^ECL2^T/S269^6.52^A. The quadruple F94^2.61^C/S188^ECL2^T/W189^ECL2^F/S269^6.52^A mutant of human melanopsin almost completely lost sensitivity to AA92593 (Fig. 5, *G* and *H*, and *I*). We note that the loss of sensitivity for AA92593 by the quadruple substitutions was not due to the substitutions-induced changes in absorption spectrum or localization in COS-1 cells. The quadruple F94^2.61^C/S188^ECL2^T/W189^ECL2^F/S269^6.52^A substitutions in human melanopsin showed little changes in absorption spectrum (Fig. 5, *J* and *K*) and GFP fluorescence (tagged on the C-terminus) signals in COS-1 cells (Fig. S10). The NanoBiT G protein dissociation assay of the F94^2.61^C/S188^ECL2^T/W189^ECL2^F/S269^6.52^A mutant also showed little sensitivity to AA92593, although a synergistic effect of W189^ECL2^F and F94^2.61^C/S188^ECL2^T/S269^6.52^A was not clearly observed (Fig. 5*L*). This is because, in the NanoBiT assay, the triple mutant F94^2.61^C/S188^ECL2^T/S269^6.52^A (without W189^ECL2^F) was already almost insensitive to AA92593 (Fig. 2*I*). In the GloSensor assay, a small difference in G protein activation between the triple and quadruple mutants in the presence of AA92593 would be amplified through the second messenger signaling cascade. In addition, we confirmed that the mouse melanopsin L207^5.42^F mutant also showed ∼26% increased susceptibility to the antagonist (Fig. 5, *M* and *N*).

Taking together the data from the GloSensor and NanoBiT assays combined with MD simulations, we concluded that amino acid residues at positions 94^2.61^, 188^ECL2^, 269^6.52^, 189^ECL2^, and 207^5.42^ in mammalian melanopsins presumably form the antagonist-binding site and play critical roles in susceptibility to AA92593. Thus, the interaction with AA92593 can be regulated by these amino acids. Next, we tested whether we could make non-mammalian melanopsins sensitive to the antagonist by the substitutions at these sites.

### Inducing the susceptibility of non-mammalian-type melanopsin (Opn4x) to AA92593 by amino acid substitutions

Our results showed that Phe-94^2.61^, Ser-188^ECL2^, Ser-269^6.52^, Trp-189^ECL2^, and Leu-207^5.42^ in mammalian melanopsins are important for susceptibility to AA92593; therefore, we attempted to make non-mammalian-type melanopsins (also known as Opn4x) susceptible to the antagonist by introducing the amino acid substitution(s). We noticed *Xenopus* Opn4x (37) and chicken Opn4-1 (38, 39) as the typical non-mammalian-type melanopsins, and we introduced Phe-94^2.61^/Ser-188^ECL2^/Ser-269^6.52^ into them (see Fig. 2*A*). We expected that the “mammalian melanopsin-type” amino acid residues would increase sensitivity of the non-mammalian-type melanopsins for the antagonist. Trp-189^ECL2^ and Leu-207^5.42^ are conserved in the non-mammalian-type melanopsins (Figs. S2 and S3). Although we did not substitute Trp-189^ECL2^, we introduced the L207^5.42^F substitution because this substitution increased susceptibility of mammalian melanopsins to the antagonist (Fig. 5, *F* and *H*, I, *M*, and *N*).

**Figure 6:**
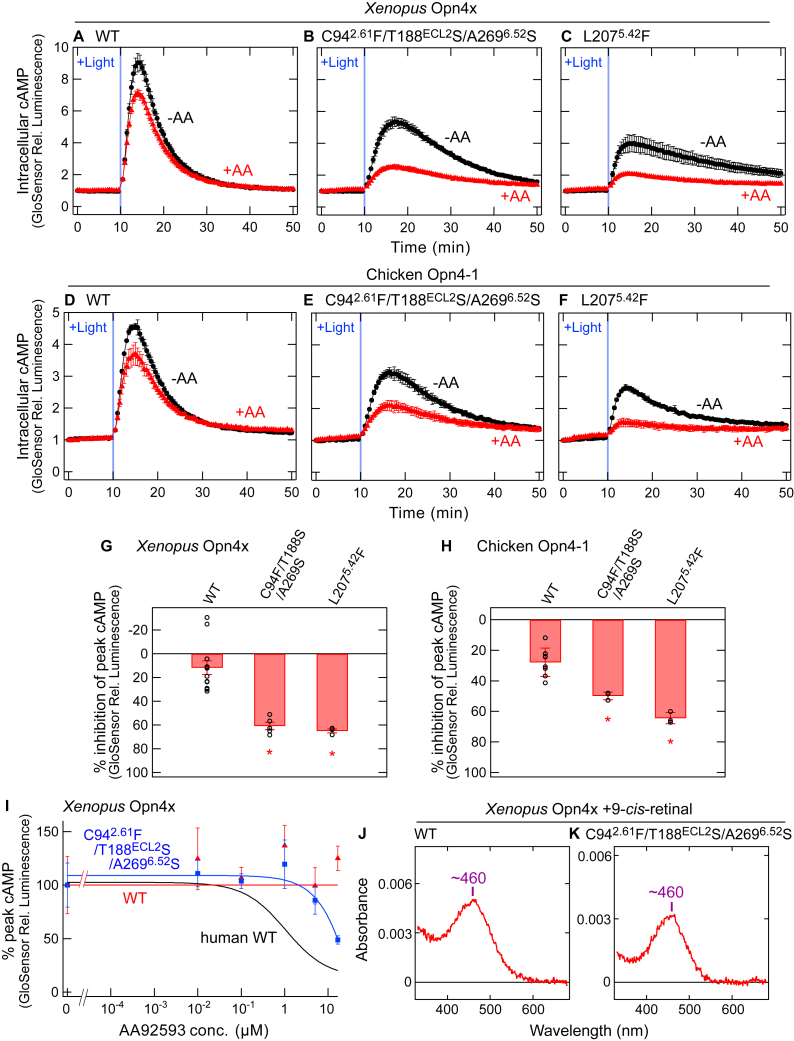
**Changes in the susceptibility of non-mammalian-type melanopsins for AA92593 by amino acid substitutions.***A*–*C*, AA92593-dependent inhibition of intracellular cAMP elevation in COS-1 cells upon Gsα/q11 activation of *Xenopus* Opn4x WT (*A*) as well as mutants C94^2.61^F/T188^ECL2^S/A269^6.52^S (*B*) and L207^5.42^F (*C*). *D*–*F*, AA92593-dependent inhibition of intracellular cAMP elevation in COS-1 cells upon Gsα/q11 activation of chicken Opn4-1 WT (*D*) as well as mutants C94^2.61^F/T188^ECL2^S/A269^6.52^S (*E*) and L207^5.42^F (*F*). In (*A*–*F*), *red* and *black* traces indicate luminescence changes in the presence and absence of 16.7 μM AA92593, respectively. *Light blue* bars indicate *white* light illumination (10 s). Luminescence levels of cAMP biosensor (GloSensor) are normalized to the values at the starting point (time = 0 min). Error bars indicate the SD values (n = 3). *G* and *H*, comparison of inhibition in peak cAMP responses by 16.7 μM AA92593 upon Gsα/q11 activation in WT and mutant of *Xenopus* Opn4x (*G*) and chicken Opn4-1 (*H*). Error bars indicate the SD values of independent experiments (n = 12, 5, and 3 for *Xenopus* Opn4x WT, C94^2.61^F/T188^ECL2^S/A269^6.52^S, and L207^5.42^F, respectively, and 8, 3, and 3 for chicken Opn4-1 WT, C94^2.61^F/T188^ECL2^S/A269^6.52^S, and L207^5.42^F, respectively). The statistical *p* values in differences from WT are 0.000076∗ and 0.00025∗ for *Xenopus* Opn4x C94^2.61^F/T188^ECL2^S/A269^6.52^S and L207^5.42^F, respectively, and 0.0027∗ and 0.000041∗ for chicken Opn4-1 C94^2.61^F/T188^ECL2^S/A269^6.52^S and L207^5.42^F, respectively (Dunnett's test following one-way ANOVA, F = 22, d. f. = 2, 17 for *Xenopus* Opn4x, F = 28, d. f. = 2, 11for chicken Opn4-1). *I*, dose-dependent reduction in peak cAMP responses upon Gsα/q11 activation by *Xenopus* Opn4x WT (*red*) and C94^2.61^F/T188^ECL2^S/A269^6.52^S mutant (*blue*). The curve of human melanopsin WT (*black*) is adopted from Figure 1*E*. Relative average peak values to the value in the absence of AA92593 are plotted against the final concentrations of AA92593. Error bars indicate the SD values (n = 3). *J* and *K*, absorption spectra of 9-*cis*-retinal bound *Xenopus* Opn4x WT (*J*) and C94^2.61^F/T188^ECL2^S/A269^6.52^S mutant (*K*). Respective λmax values are indicated.

The GsX GloSensor assay using Gsα/q11 of *Xenopus* Opn4x and chicken Opn4-1 detected the light-dependent Gq activation in both non-mammalian-type melanopsins (Fig. 6, *A* and *D*). Unlike mammalian melanopsins (Fig. 1), the addition of AA92593 to *Xenopus* Opn4x and chicken Opn4-1 did not effectively inhibit the light-dependent responses (Fig. 6, *A* and *D*, and *I*). The ineffectiveness of AA92593 is consistent with the fact that Phe-94^2.61^, Ser-188^ECL2^, and Ser-269^6.52^ are not conserved among non-mammalian-type melanopsins (Figs. 2*A*, S2 and S3). If these three residues are important for the antagonistic effect against melanopsins, the introduction of Phe-94^2.61^/Ser-188^ECL2^/Ser-269^6.52^ into the non-mammalian-type melanopsins would make them susceptible to AA92593. As expected, the substitutions of C94^2.61^F/T188S^ECL2^/A269^6.52^S in *Xenopus* Opn4x and chicken Opn4-1 increased their sensitivity to AA92593 by ∼49% and ∼22%, respectively (Fig. 6, *B* and *E*, G–*I*). Based on the loss-of-function properties of F94^2.61^C/S188^ECL2^T/S269^6.52^A mutants of mammalian-type melanopsins and the gain-of-function properties of C94^2.61^F/T188^ECL2^S/A269^6.52^S mutants of non-mammalian-type melanopsins (Figs. 2*G*, 3*E*, 6, *G* and *H*), we propose that Phe-94^2.61^, Ser-188^ECL2^, and Ser-269^6.52^ are required for specific interactions with AA92593 as an effective antagonist. We note that the C94^2.61^F/T188^ECL2^S/A269^6.52^S substitutions on *Xenopus* Opn4x did not cause a large shift in its absorption spectrum or obvious changes in localization of GFP fluorescence (tagged on the C-terminus) in COS-1 cells (Figs. 6, *J* and *K*, and S10).

Similar to human and mouse melanopsins (Fig. 5, *F* and *H*, I, *M*, and *N*), the introduction of a Phe residue at position 207^5.42^ in *Xenopus* Opn4x and Chicken Opn4-1 increased their susceptibility of the melanopsins for AA92593 by ∼53% and ∼37%, respectively, although the substitutions reduced light-dependent cAMP responses by the melanopsins (Fig. 6, *C* and *F*, G and *H*). The results of substitutions at position 207^5.42^ supported the idea that AA92593 binds to a similar site in both non-mammalian-type and mammalian-type melanopsins, and substitutions at positions 94^2.61^, 188^ECL2^, 269^6.52^, and 207^5.42^ make the non-mammalian-type melanopsins interact with the antagonist in a similar way to AA92593-sensitive mammalian-type melanopsins.

### Inducing the susceptibility of invertebrate melanopsin to AA92593 by amino acid substitutions

**Figure 7:**
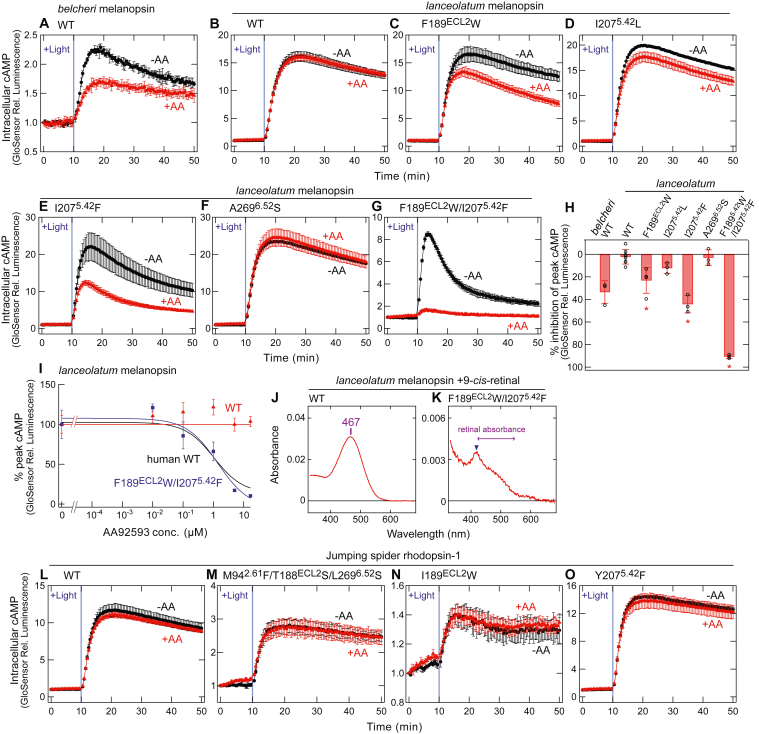
**Changes in the susceptibility of invertebrate melanopsins and a non-melanopsin Gq-coupled opsin for AA92593 by amino acid substitutions.***A*, AA92593-dependent inhibition of intracellular cAMP elevation in COS-1 cells upon Gsα/q11 activation of *belcheri* melanopsin WT. Error bars indicate the SD values (n = 3). *B*–*G*, AA92593-dependent inhibition of intracellular cAMP elevation in COS-1 cells upon Gsα/q11 activation of *lanceolatum* melanopsin WT (*B*) as well as mutants F189^ECL2^W (*C*), I207^5.42^L (*D*), I207^5.42^F (*E*), A269^6.52^S (*F*), and F189^ECL2^W/I207^5.42^F (*G*). Error bars indicate the SD values (n = 3). *H*, comparison of inhibition in peak cAMP responses by 16.7 μM AA92593 upon Gsα/q11 activation in *belcheri* melanopsin WT and *lanceolatum* melanopsin WT and mutants. Error bars indicate the SD values of independent experiments (n = 3, 8, 4, 3, 3, 3, and 3 for *belcheri* WT, *lanceolatum* WT, F189^ECL2^W, I207^5.42^L, I207^5.42^F, A269^6.52^S, F189^ECL2^W/I207^5.42^F, respectively). The statistical *p* values in differences from WT are <0.001∗, 0.23, <0.001∗, 1.00, and <0.001∗ for F189^ECL2^W, I207^5.42^L, I207^5.42^F, A269^6.52^S, F189^ECL2^W/I207^5.42^F, respectively (Dunnett's test following one-way ANOVA, F = 77, d. f. = 5, 18). *I*, dose-dependent reduction in peak cAMP responses upon Gsα/q11 activation by *lanceolatum* melanopsin WT (*red*) and F189^ECL2^W/I207^5.42^F mutant (*blue*). The curve of human melanopsin WT (*black*) is adopted from Figure 1*E*. Relative average peak values to the value in the absence of AA92593 are plotted against the final concentrations of AA92593. Error bars indicate the SD values (n = 3). Fitting parameters for the F189^ECL2^W/I207^5.42^F mutant: *max*, 107.65 ± 12.6; *min*, −5.22 ± 4.53; *rate*, −0.84 ± 0.75. IC_50_ values for the F189^ECL2^W/I207^5.42^F mutant is 1.40 ± 0.88 μM. *J* and *K*, absorption spectra of 9-*cis*-retinal bound *lanceolatum* melanopsin WT (*J*) and F189^ECL2^W/I207^5.42^F mutant (*K*). λmax value of WT is indicated. Note that the sharp abruption peak at ∼420 nm in (*K*) (*blue* arrowhead) is caused by contamination with cytochrome, not by retinal bound to melanopsin (*purple* arrow). *L*–*O*, AA92593-dependent inhibition of intracellular cAMP elevation in COS-1 cells upon Gsα/q11 activation of jumping spider rhodopsin-1 WT (*L*), M94^2.61^F/T188^ECL2^S/L269^6.52^S (*M*), I189^ECL2^W (*N*), and Y207^5.42^F (*O*). Error bars indicate the SD values (n = 3). In (*A*–*G*) and (*L*–*O*), *red* and *black* traces indicate luminescence changes in the presence and absence of 16.7 μM AA92593, respectively. *Light blue* bars indicate *white* light illumination (10 s). Luminescence levels of cAMP biosensor (GloSensor) are normalized to the values at the starting point (time = 0 min).

Non-mammalian-type melanopsins were successfully converted into AA92593-susceptible types by introducing Phe-94^2.61^/Ser-188^ECL2^/Ser-269^6.52^ or Phe-207^5.42^ (Fig. 6). We attempted to extend the functional conversion targets to invertebrate melanopsin (see Fig. S2). Cephalochordate amphioxus species *Branchiostoma belcheri* and *Branchiostoma lanceolatum* possess melanopsin (16, 40), and in this study, we indicate these melanopsins as *belcheri* and *lanceolatum* melanopsins, respectively. We assessed the susceptibility of *belcheri* and *lanceolatum* melanopsins to AA92593 using the GsX GloSensor assay with Gsα/q11. In the absence of AA92593, the *belcheri* melanopsin produced smaller light-dependent cAMP responses than the other melanopsins assessed in this study (Fig. 7*A*), whereas the *lanceolatum* melanopsin produced robust cAMP responses upon light illumination (Fig. 7*B*). Surprisingly, light-induced cellular responses of the *belcheri* melanopsin were reduced by ∼34% by the addition of AA92593 (Fig. 7*A*). On the other hand, responses by the *lanceolatum* melanopsin were insensitive to the antagonist (Fig. 7*B*), similar to non-mammalian-type melanopsins (Fig. 6, *A* and *D*). Although the mechanism underlying the AA92593 susceptibility of *belcheri* melanopsin is unknown, we attempted to convert AA92593-insensivite *lanceolatum* melanopsin to a susceptible type *via* amino acid substitutions.

Because *lanceolatum* melanopsin endogenously possesses Phe-94^2.61^ and Ser-188^ECL2^ (Fig. 2*A*), we substituted Ala-269^6.52^ with Ser, Phe-189^ECL2^ with Trp, and Ile-207^5.42^ with Leu or Phe in the invertebrate melanopsin. We assessed the effect of the antagonist on the mutants using the GsX GloSensor assay with Gsα/q11. F189^ECL2^W and I207^5.42^F substitutions significantly increased the sensitivity to AA92593 by ∼21% and ∼42%, respectively (Fig. 7, *C,* *E*, and *H*), whereas I207^5.42^L and A269^6.52^S caused little changes (Fig. 7, *D,* *F*, and *H*). Furthermore, the double substitution F189^ECL2^W/I207^5.42^F further increased the susceptibility of the *lanceolatum* melanopsin to the antagonist by ∼88% (Fig. 7, *G,* *H*, and *I*). The AA92593-induced inhibition of the double mutant F189^ECL2^W/I207^5.42^F was as efficient as that of human melanopsin (Fig. 7*I*). We also confirmed that the F189^ECL2^W/I207^5.42^F substitutions did not cause obvious changes in the localization of the GFP-tagged melanopsin proteins in COS-1 cells (Fig. S10). Also, the F189^ECL2^W/I207^5.42^F mutant showed a retinal absorbance at around 400 to 500 nm regions, which was not largely different from the WT's spectrum, although the yield of the mutant after purification was quite lower than that of WT (Fig. 7, *J* and *K*). Taken together, the introduction of Trp-189^ECL2^ and Phe-207^5.42^ rendered *lanceolatum* melanopsin highly sensitive to the antagonist (Fig. 7, *G,* *H*, and *I*).

We next tested the effect of AA92593 on jumping spider rhodopsin-1, a Gq-coupled visual opsin but not melanopsin (see Figs. 2, S2, and S3) (41). Similar to non-mammalian-type and invertebrate melanopsins, jumping spider rhodopsin-1 was not sensitive to AA92593 as assessed using the GsX GloSensor assay (Fig. 7*L*). The introduction of Phe-94^2.61^/Ser-188^ECL2^/Ser-269^6.52^, Trp-189^ECL2^, or Phe-207^5.42^ into jumping spider rhodopsin-1 did not increase its sensitivity to AA92593 (Fig. 7, *M*–*O*), unlike in the cases of non-mammalian-type and invertebrate melanopsins (Figs. 6 and 7). The introduction of I189^ECL2^W/Y207^5.42^F impaired the light-dependent cAMP responses even in the absence of AA92593 (Fig. S11). Taken together, the non-melanopsin Gq-coupled opsin cannot be functionally converted to an AA92593-sensitive type by site-directed mutagenesis, in contrast to non-mammalian-type and invertebrate melanopsins.

## Discussion

In the present study, we investigated the molecular mechanisms underlying the antagonistic effects of AA92593 on mammalian melanopsins. Our experiments indicated that AA92593 is specifically effective on mammalian melanopsins, and combination of cell-based assays and computational simulations identified five amino acid residues which comprise the antagonist-binding site and are responsible for the specificity. Based on these results, we discuss how AA92593 acts as the antagonist specifically against mammalian melanopsins. We propose that the insights gained from this study can be applied to further physiological studies.

### Molecular basis underlying the specificity between mammalian melanopsin and AA92593

Our site-directed mutagenesis data showed that amino acid substitutions at positions 94^2.61^, 188^ECL2^, 269^6.52^, 189^ECL2^, and 207^5.42^ decreased or increased the antagonistic effects of AA92593 (Figure 2, Figure 3 and Figure 2, Figure 3). Our docking and MD simulations of human melanopsin revealed that these amino acids are located at the AA92593-binding site (Fig. 4). The most effective single substitution site reducing the effect of AA92593 on mammalian melanopsin was Trp-189^ECL2^, which is conserved among mammalian-type and non-mammalian-type melanopsins (see Fig. S2). In the simulated structural models of human melanopsin (Fig. 4), Trp-189^ECL2^ was found to interact with the antagonist from the extracellular side, primarily *via* van der Waals interactions (Figs. 4*D* and S7*A*). These consistent experimental and simulation results suggest that these five amino acid residues modulate the antagonistic effects through direct interactions. This interpretation is supported by the fact that the introduction of “mammalian melanopsin-type” amino acid residues (Phe-94^2.61^, Ser-188^ECL2^, and Ser-269^6.52^) as well as Trp-189^ECL2^ and Phe-207^5.42^ into non-mammalian and invertebrate melanopsins made them more susceptible to the antagonist (Figs. 6 and 7).

Substitutions at the five sites converted AA92593-insensitive melanopsins such as *Xenopus* Opn4x and *lanceolatum* melanopsin to sensitive ones (Figs. 6 and 7) but did not convert jumping spider rhodopsin-1, a non-melanopsin Gq-coupled opsin (Fig. 7, *L–O*). These results suggest that the molecular architecture around the AA92593-binding site is somewhat different between melanopsins and other Gq-coupled opsins, although the overall structure is highly conserved among opsins including melanopsins. In order to design chemicals that selectively modulate activities of melanopsins or other opsins, the small static and/or dynamic structural differences should be considered. To do so, combination of experiments, molecular simulations, and structure predictions would be a powerful strategy.

Several class A GPCRs including some opsins are reported to form oligomers (42), but the effect of oligomerization on their functionalities is limited (43, 44, 45). Since the sites 94^2.61^, 188^ECL2^, 269^6.52^, 189^ECL2^, and 207^5.42^ are located inside the melanopsin molecules (see Fig. 4, *A* and *B*), substitutions at these sites would not affect the oligomerization of melanopsins.

In addition to the five amino acid residues, Trp-265^6.48^ was also found to be in contact with the antagonist from the intracellular side and showed strong binding. Because Trp-265^6.48^ is highly conserved in GPCRs and is expected to be important for their function (33, 36, 46), this strong interaction between AA92593 and Trp-265^6.48^ may play a role in suppressing the activation of human melanopsin.

### Modulation of AA92593 sensitivity in melanopsins for physiological studies

Our data clearly indicated that the susceptibility to AA92593 as an antagonist can be modulated by substituting amino acid residues in a wide variety of melanopsins. Substitutions at positions 94^2.61^/188^ECL2^/269^6.52^/189^ECL2^/207^5.42^ decreased or increased the susceptibility of mammalian and other melanopsins. Most mammals, including humans and mice, possess a single melanopsin gene (opn4m) in their genomes (39), and the Opn4m primarily functions in ipRGCs. In contrast, many non-mammalian vertebrates possess multiple melanopsin genes (opn4m and opn4x). Melanopsins of non-mammalian vertebrates are expressed in various cells, including ipRGCs, but the physiological role of each melanopsin remains unclear (39, 47, 48). Our results showed that AA92593 is more effective against mammalian-type melanopsin (Opn4m) rather than against non-mammalian-type one (Opn4x) and that the effectiveness of AA92593 on a specific melanopsin can be more accurately predicted based on the amino acid sequence at the five sites (Fig. S2). In addition, susceptibility of invertebrate melanopsins to AA92593 was dramatically changed by the introduction of Trp-189^ECL2^ and Phe-207^5.42^ (Fig. 7, *G*–*I*).

Recent progress in genome editing techniques (49) has enabled the elimination of a specific melanopsin gene in animals, but temporal silencing of specific melanopsin functions remains difficult. Genome editing to introduce amino acid substitutions at positions 94^2.61^/188^ECL2^/269^6.52^/189^ECL2^/207^5.42^ into a specific melanopsin can increase or decrease the susceptibility of the target melanopsin to AA92593. In particular, the introductions of Trp-189^ECL2^ or Phe-207^5.42^ were the most effective single substitutions to increase AA92593 antagonism (Figs. 6 and 7). Such a gene manipulation to introduce amino acid residue(s) that modulate the effect of AA92593 will enable the antagonist-mediated suppression of the targeted melanopsin functions. The combination of site-directed genome editing and AA92593 administration in living animals could silence specific melanopsin functions with high spatiotemporal resolution.

## Experimental procedures

### Constructs

In the constructs for melanopsin expression (opsin name - GenBank ID: human melanopsin - NM_033282.4, mouse melanopsin - NM_013887.2, *Xenopus* Opn4x - NM_001085674.1, chicken opn4-1 - NM_001397961.1, *B. belcheri* melanopsin - AB205400.1, and *B. lanceolatum* melanopsin - MF464477.1), the C-terminal amino acid residues (94, 140, 200, 214, 296, and 308 residues for human (9), mouse (9), *Xenopus*, chicken, *belcheri* (16), and *lanceolatum* melanopsins, respectively) were removed, the 1D4 tag sequence (ETSQVAPA) was added, and inserted into the EcoRI/NotI site in a mammalian expression vector pMT. Jumping spider rhodopsin-1 (GenBank ID: AB251846.1) was also added with the 1D4 tag and inserted into pMT vector. Human and mouse melanopsins, and jumping spider rhodopsin-1 in pCDNA3.1 were kindly provided from Drs. Akihisa Terakita and Mitsumasa Koyanagi (Osaka Metropolitan University). In this paper, each opsin with the C-terminal sequence modifications as described above is denoted “WT.” Bovine Gsα and its mutants Gsα/q11 and Gsα/i11 (C-terminal 11 residues are replaced with Gqα and Giα, respectively) were also inserted into the pMT vector. The GFP-tagged melanopsin constructs, in which EGFP protein fused with C-termini of melanopsins, were prepared as previously described (9, 50). As a control, mouse TWIK1, a potassium channel, with the GFP-tag on C-terminus was also prepared (50), because the channel is known to be mainly located in intracellular compartments (51). The expression vector pGlo-22F for GloSensor assay was purchased from Promega. For NanoBiT assay, the coding sequences of Lg-BiT inserted human Gqα, human Gβ1, and the Sm-BiT-fused human Gγ2 (C68S), RIC8A were constructed according to refs. (23, 24), and inserted into the pMT vector. The expression vector for RIC8A was purchased from GenScript and subcloned into the pMT vector.

To construct the various melanopsin mutants, Gsα/q11, Gsα/i11, and Gqα/R183Q-LgBiT mutations were introduced into the cDNA sequence by PCR reaction. The sequences were confirmed by DNA sequencing. The plasmid DNA used for transfection was prepared using either the FastGene Plasmid Mini Kit (Nippon Genetics) or NucleoBond Xtra Midi/Maxi (TAKARA).

### Transfection to COS-1 cells for GloSensor assays, NanoBiT G protein dissociation assay, GFP-fluorescence microscopy, and melanopsin purification

For GloSensor assay, opsins, GloSensor assay sensor (coded by pGlo-22F), and NanoBiT-tagged G proteins were transiently expressed in COS-1 cells (kindly provided from Dr David Farrens, Oregon Health and Science University) using PEI as described previously (24, 52). For the GsX GloSensor assay (12, 13), each well of a 96-well assay plate (Corning) was transfected with 50 ng opsin plasmid, 16.7 ng G protein plasmid, 50 ng pGlo-22F plasmid (Promega), and 500 ng PEI in 25 μl Opti-mem and 75 μl Dulbecco's modified Eagle's medium (D-MEM). For NanoBiT G protein dissociation assay, each well of a 96-well assay plate was transfected with 50 ng opsin plasmid, 5 ng Lg-BiT inserted Gqα (Gqα-LgBiT) plasmid, 25 ng Gβ1 plasmid, 25 ng Sm-BiT fused Gγ2 plasmid, 5 ng RIC8A plasmid, and 500 ng PEI in 25 μl Opti-mem and 75 μl D-MEM. For GFP-fluorescence microscopy, a 35-mm cell culture dish (IWAKI) was transfected with 2 μg melanopsin-GFP plasmid and 8 μg PEI in 400 μl Opti-mem and 1.2 ml D-MEM. For melanopsin purification, ten of 100-mm cell culture dishes (NEST) were transfected with 150 μg melanopsin plasmid and 500 μg PEI in 25 ml Opti mem and 75 ml D-MEM.

### Fluorescence microscopy for GFP-tagged melanopsins

COS-1 cells transfected with pMT vectors coding GFP-tagged melanopsin constructs were incubated in CO_2_ incubator for 1 day, and the media were exchanged with PBS buffer to reduce autofluorescence from the medium. The GFP fluorescence in the unfixed cells was analyzed using a fluorescence microscope CKX53 and a LED light source U-LGPS (Olympus), equipped with GFP fluorescence detection filters (excitation: 480 nm, emission: 530 nm).

### Melanopsin purification and UV-Vis spectroscopy

The transfected cells were harvested 48 h after transfection as described previously (9). The collected cells were incubated with 9-*cis*-retinal overnight, and membrane proteins were solubilized with 1.25% DDM (Dojindo), 20 mM Hepes, 140 mM NaCl, 0.25% cholesterol hemisuccinate (Sigma-Aldrich) 25 mM Tris, 10% glycerol, pH 7.0. The solubilized materials were mixed with 1D4-agarose overnight, and the mixture was transferred into Bio-Spin columns (Bio-rad). The columns were washed with 0.05% DDM, 2 mM ATP, 1 M NaCl, 3 mM MgCl_2_, 0.01% cholesterol hemisuccinate, 1 mM Tris, 10% glycerol in PBS, and subsequently washed with 0.05% DDM, 140 mM NaCl, 0.01% cholesterol hemisuccinate, 1 mM Tris, 10% glycerol, 20 mM Hepes, pH 7 (buffer A). The 1D4-tagged pigments were eluted with buffer A containing 0.45 mg/ml 1D4 peptide (TETSQVAPA) (TOYOBO). Absorption spectra of purified melanopsins were recorded with a Shimadzu UV-2600 spectrophotometer (Shimadzu). The samples were kept at 10 °C.

### GsX GloSensor assay

The transfected COS-1 cells were incubated at 37 °C, 5% CO_2_ for 2 days, and the medium was aspirated and exchanged with 50 μl of HBSS (145 mM NaCl, 10 mM D-glucose, 5 mM KCl, 1 mM MgCl_2_, 1.7 mM CaCl_2_, 1.5 mM NaHCO_3_, 10 mM Hepes, pH 7.4), containing 33.3, 20, 10, 2, 0.2, 0.02, 0.002, or 0 μM AA92593, 0.5% dimetyl sulfoxide (DMSO), and 4% (vol/vol) GloSensor cAMP reagent stock solution. Under our experimental conditions, >50 μM AA92593 was not fully dissolved in the HBSS solution, and we set maximal concentration of AA92593 (Glixx Laboratories) at 33.3 μM (final concentration of 16.7 μM, see below). Then, the cells were incubated at room temperature for 1 h, and luminescence changes were monitored. After incubation of the cells with AA92593, luminescence measurement was interrupted, the plate was ejected, and the cells were added with 50 μl of HBSS containing 0.2 μM 9-*cis*-retinal. The final exogenous AA92593 concentrations were 16.7, 10, 5, 1, 0.1, 0.01, 0.001, or 0 μM, and retinal concentration was 0.1 μM. Twenty millimolars of stock solution of AA92593 was prepared in DMSO, and final concentration of DMSO was kept at 0.25% regardless final AA92593 concentration. Then, the cells were incubated at room temperature for 1 h. Luminescence was measured using GM-2000 or GM-3510 microplate reader (Promega). Luminescence level of each well was measured every 30 s with integration time of 0.6 to 0.9 s. To illuminate opsins, luminescence measurement was interrupted, the plate was ejected, and the plate was illuminated by white light with CN-160 LED video light (light intensity, ∼9 mW/cm^2^) (NEEWER). In Figure 1*A*, light intensity was varied to obtain the light intensity—GloSensor signal relationship. After illumination, luminescence measurement was resumed. The measured luminescence levels were normalized to the level at the starting point (time = 0 min). To calculate dose-response curve of light stimulation or AA92593, we fitted data with the Hill equation {y = *base* + (*max* – *base*)/(1 + [IC_50_/x]^*rate*^} [y, relative GloSensor signal intensity; x, light intensity or AA92593 concentration; *base*, *max*, and *rate* are fitting parameters] using Igor Pro (WaveMetrics). In each figure legend, the calculated three parameters and EC_50_ or IC_50_ value are indicated.

### NanoBiT G protein dissociation assay

The transfected COS-1 cells were incubated at 37 °C, 5% CO_2_ for 1 day, and the medium was aspirated, followed by the addition of 50 μl of HBSS containing 33.3, 3.33, or 0 μM AA92593, 0.5% DMSO, and 10 μM coelenterazine h (Wako). Then, the cells were incubated at room temperature for 1 h. After incubation of the cells, luminescence measurement was interrupted, the plate was ejected, and the cells were added 50 μl of HBSS containing 0.2 μM 9-*cis*-retinal. The final concentration of AA92593 were 16.7, 1.67, or 0 μM (DMSO concentration of 0.25%), and retinal concentration was 0.1 μM. Then, the cells were incubated at room temperature for 1 h. Luminescence was measured using GM-2000 or GM-3510 microplate reader (Promega). Luminescence level of each well was measured every 18 s with integration time of 0.3 s. To illuminate opsins, luminescence measurement was interrupted, the plate was ejected, and the plate was illuminated by white light with CN-160 LED video light (light intensity, ∼9 mW/cm^2^) (NEEWER). After illumination, luminescence measurement was resumed. The measured luminescence levels were normalized to the level at the starting point (time = 0 min).

### Docking and MD simulations

The predicted structure of the human melanopsin was taken from the AlphaFold Protein Structure Database (ID: Q9UHM6). Disordered part of the protein was removed, and the residues 65 to 373 out of the total 478 residues of the human melanopsin were used in all of the current calculations. The structure of AA92593 was built by GaussView (53). Docking calculations of the binding of AA92593 to the human melanopsin was performed using AutoDock Vina (25) and DiffDock (27). AutoDock Tools 1.5.4 (54) was used to set up the protein and ligand for AutoDock by deleting the water molecules and adding hydrogen atoms. Rigid-body docking was performed using AutoDock with the query box of 47.25 Å × 38.25 Å × 47.25 Å centered at (−7.75, 1.39, −0.12) to cover the expected binding pocket. Twenty binding poses (num_modes = 20) were requested, and the exhaustiveness parameter was set to 80. For DiffDock, the default parameters with inference_steps = 20, samples_per_complex = 40, and batch_size = 10 were used. The melanopsin–AA92593 complex with the best score from DiffDock was picked for the structural analysis and the initial structure for the subsequent MD simulations. The initial structure for the simulation containing lipid and water molecules were prepared using the CHARMM-GUI Membrane Builder (55). The membrane was composed of 100% POPC with the KCl salt concentration of 150 mM, and the system size was 80.3 Å × 80.3 Å × 125.2 Å. The CHARMM36 m force field parameters were used for the proteins, the ligand force field was set up using the CHARMM General Force Field (CGenFF), and the waters were treated with the TIP3P water model. The long-range electrostatic interactions were treated by the particle mesh Ewald method, whereas short-range nonbonded interactions were cut off at 12 Å and 10 Å during equilibration and production runs, respectively. Energy minimization with the steepest descent method was performed for 5000 steps. A series of short equilibrations were performed by gradually releasing the restraints on the position and dihedral of protein and membrane during a total of 1.875 ns by following the script generated by CHARMM-GUI. During these equilibration steps, the time step in MD simulation was increased from 1 to 2 fs, and position restraints on the lipid headgroups were relaxed. Subsequently, all position and dihedral restraints were removed, and 10 ns simulation under the constant-NPT condition was performed to complete the equilibration. The Langevin thermostat and the Berendsen barostat were used to maintain the temperature at 303.15 K and pressure at 1 bar, respectively. A 1μs MD simulation under the constant-NPT condition at 303.15 K and 1 bar was performed for production. To elucidate the key residues which have stronger influence on the binding free energy, per-residue decompositions of the MMPBSA binding free energy were performed using AmberTools (56). The last 300 ns (3000 frames) of the 1μs trajectory were used to calculate the binding free energy. In MMPBSA, the membrane was modeled as a solid slab of 34.25 Å centered at a Z-offset of 5.0 Å. The dielectric constant of the membrane, protein, and water phases were set to 4.0, 20.0, and 80.0, respectively. The water phase ionic strength was set to be 150 mM. The per-residue energy contributions of the individual amino acids to the total MMPBSA energy were calculated to identify the amino acids residues participating in the binding. Here, the negative sign of the decomposed binding free energy contribution indicates that the residue stabilizes binding and thus contributes to strengthen binding affinity. All molecular dynamics calculations were performed using Amber 22 software package (57).

## Data availability

All data are contained in the manuscript.

## Supporting information

This article contains supporting information.

## Conflicts of interests

The authors declare that they have no conflicts of interests with the contents of this article.
