# Supplementary material for: Molecular basis underlying the specificity of an antagonist AA92593 for mammalian melanopsins
Source: J Biol Chem. 2025 Mar 26;301(5):108461. doi: 10.1016/j.jbc.2025.108461 (PMC12051620; doi:10.1016/j.jbc.2025.108461)
Supplement: Supplementary Figures [file mmc1.pdf]

Supporting Information

Molecular basis underlying the specificity of an antagonist AA92593 for mammamalian melanopsins

Kohei Obayashi[1], Ruisi Zou[2], Tomoki Kawaguchi[1], Toshifumi Mori[2, 3], Hisao Tsukamoto[1, 4*]

1 Department of Biology, Graduate School of Science, Kobe University, Kobe, Japan, 2 Interdisciplinary Graduate School of Engineering Sciences, Kyushu University, Fukuoka, Japan, 3 Institute for Materials Chemistry and Engineering, Kyushu University, Fukuoka, Japan, 4 Center of Optical Scattering Image Science, Kobe University, Japan.

Correspondence should be addressed to Hisao Tsukamoto; Department of Biology, Kobe University, 1-1, Rokkodai-cho, Nada-Ku, Kobe, 657-8501, Japan; tsukamoh@people.kobe-u.ac.jp

Supplemental Figure legends

<u>Fig. S1</u> AA92593-dependent inhibition of intracellular cAMP elevation in COS-1 cells upon activation of endogenous G proteins, exogenous Gsα, and its mutants Gsα/q11 and Gsα/i11 by human melanopsin.

Representative raw luminescence changes upon activation of endogenous G proteins (*A*), exogenous Gsα/q11 (*B*), Gsα (*C*), and Gsα/i11 (*D*) by human melanopsin. Note that absolute luminescence level was much larger in activation of exogenous Gsα or its mutants, probably due to the high expression levels of these exogenous proteins.

<u>Fig. S2</u> Comparison of amino acid residues in the retinal-binding site of various melanopsins and other opsins.

Amino acid residues in the putative retinal-binding site of Opn4m, Opn4x, invertebrate melanopsin, non-melanopsin Gq-coupled opsin, bovine rhodopsin (amino acid number is based on bovine rhodopsin sequence and Ballesteros/Weinstein (GPCRdb) numbering) are shown, and schematic phylogenetic relationship of the opsins was also indicated.

<u>Fig. S3</u> Amino acid sequence alignment of melanopsins and other opsins used in this study.

Amino acid sequences of human melanopsin, mouse melanopsin, *Xenopus* Opn4x, chicken Opn4-1, *belcheri* melanopsin, *lanceolatum* melanopsin, jumping spider rhodopsin-1, and bovine rhodopsin are shown. Putative regions corresponding to transmembrane helices are indicated. Amino acid residues at positions $94^{2.61}$, 188^{ECL2}, 189^{ECL2}, $207^{5.42}$, and $269^{6.52}$ are shown (red), and the "standard" residues (number 50 in each transmembrane helix) in Ballesteros/Weinstein (GPCRdb) numbering are also shown (blue). Note that the C-terminal amino acid residues of melanopsins (94, 140, 200, 214, 296, and 308 residues for the human, mouse, *Xenopus*, chicken, *belcheri*, and *lanceolatum* melanopsins, respectively) were removed (see "Experimental Procedures").

Fig. S4 **Comparison of inhibition in peak cAMP responses by 16.7 μM AA92593 upon Gsα/q11 activation in human melanopsin WT (*A*) and F94$^{2.61}$C mutant (*B*) in 3 times-repeated experiments.**

Note that in each experiment, F94$^{2.61}$C showed a less AA92593-induced suppression, although the difference was not statistically significant (see main text).

Fig. S5 **NanoBiT Gq dissociation assay using Gqα-LgBiT or Gqα/R183Q-LgBiT on human melanopsin WT.**

Red and blue traces indicate luminescence changes using Gqα-LgBiT and Gqα/R183Q-LgBiT, respectively, in the absence of AA92593. Light blue bars indicate white light illumination (10 sec). NanoLuc luminescence levels are normalized to the values at the starting point (time = 0 min). Error bars indicate the SD values (n = 3).

Fig. S6 **Docking structure of human melanopsin-AA92593 complex predicted using AutoDock.**

Location of predicted binding positions of AA92593 from AutoDock. The protein structure of the human melanopsin was taken from AlphaFold Protein Structure Database (ID: Q9UHM6, residues 65 to 373). Colors of the ligand indicate atom types, and the top 20 best scored predicted positions of ligands are superimposed.

Fig. S7 **Energy decomposition of the per-residue decomposition for the binding of AA92593 to human melanopsin.**

Van der Waals (*A*) and electrostatic (*B*) contributions to the per-residue decomposition of the binding energy for the binding of AA92593 to human melanopsin calculated from the last 300 ns of the MD simulation trajectory using MMPBSA.

Fig. S8 **Changes of distances between AA92593 and selected residues in human melanopsin during MD simulation.**

Time evolution of the distances between AA92593 and selected residues in human melanopsin along the 1 μs MD trajectory of the complex. The results for Ile-122$^{3.37}$, Trp-189^{ECL2}, Leu-207$^{5.42}$, Phe-212$^{5.47}$, Trp-265$^{6.48}$, and Lys-296$^{7.43}$ are shown.

<u>Fig. S9 Hydrogen bond network about the ligand involving water in the binding pocket.</u>

(*A*) Hydrogen bond network typically found in the binding pocket of human melanopsin (taken from the snapshot at 720 ns). (*B*) Radial distribution functions between O1 and O2 of the SO_2 group in AA92593 and hydrogen atoms in water, calculated from 0.7 to 1 μs of the MD trajectory.

<u>Fig. S10</u> GFP fluorescence images of GFP-tagged human melanopsin, *Xenopus* Opn4x, *lanceolatum* WTs and mutants.

GFP fluorescence (green) images of unfixed COS-1 cells expressing GFP-tagged human melanopsin WT (*A*), its F94$^{2.61}$C/S188^{ECL2}T/W189^{ECL2}F/S269$^{6.52}$A mutant (*B*), *Xenopus* Opn4x (*C*), its C94$^{2.61}$F/T188^{ECL2}S/A269$^{6.52}$S mutant (*D*), *lanceolatum* melanopsin WT (*E*), its F189^{ECL2}W/I207$^{5.42}$F mutant, and a potassium channel TWIK1 (*F*) are shown. The image of TWIK1 is shown as a control, because the channel is known to be mainly located in intracellular compartments. Panels *A-E* indicate the amino acid substitutions did not largely affect localization of respective melanopsin molecules in COS-1 cells. Scale bar (50 μm) is indicated in each panel. See main text for detail.

<u>Fig S11</u> AA92593-dependent inhibition of intracellular cAMP elevation in COS-1 cells upon Gsα/q11 activation of jumping spider rhodopsin-1 I189^{ECL2}W/Y207$^{5.42}$F mutant. Red and black traces indicate luminescence changes in the presence and absence of 16.7 μM AA92593, respectively. Light blue bars indicate white light illumination (10 sec). Luminescence levels of cAMP biosensor (GloSensor) are normalized to the values at the starting point (time = 0 min). Error bars indicate the SD values (n = 3).

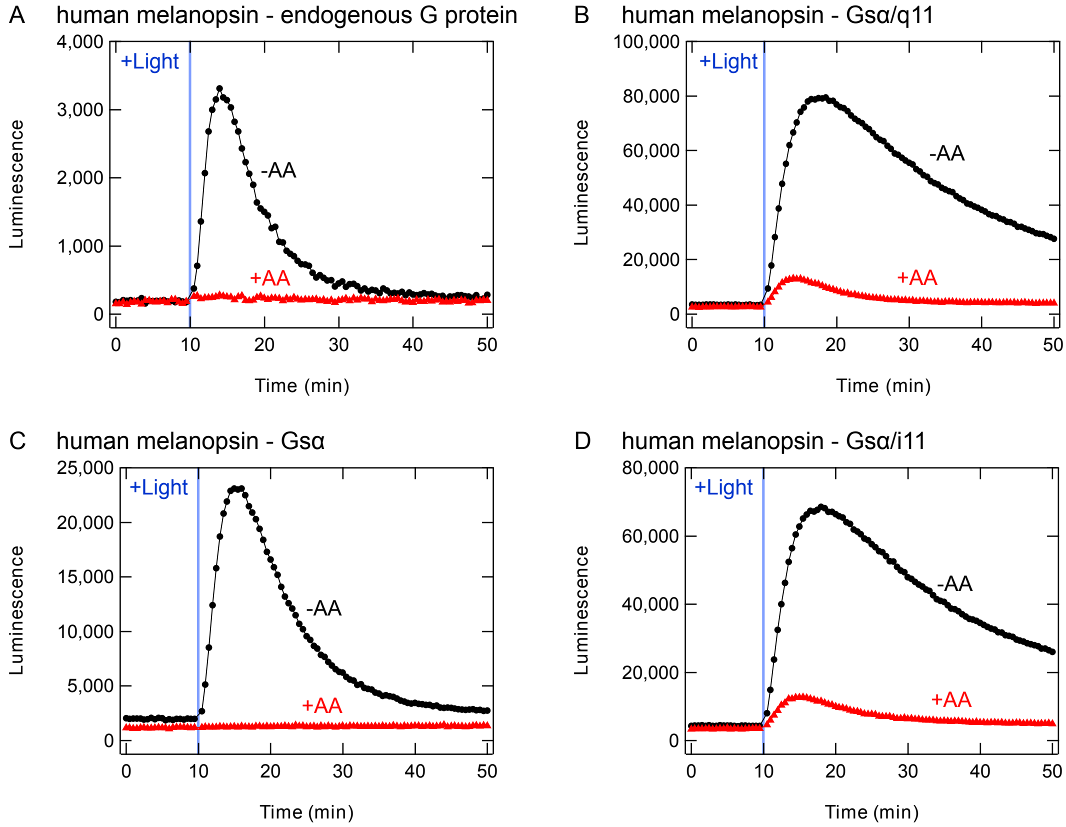

Supplemental Fig. S1 Obayashi et al.

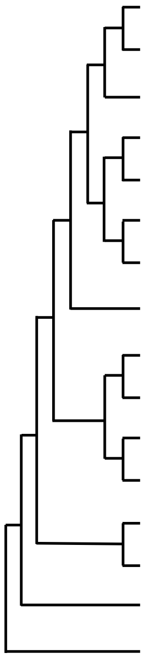

	86<br>2.53	89<br>2.56	90<br>2.57	94<br>2.61	113<br>3.28	117<br>3.32	118<br>3.33	121<br>3.36	122<br>3.37	164<br>4.53	181<br>ECL2	186<br>ECL2	188<br>ECL2	189<br>ECL2	207<br>5.42	208<br>5.43	211<br>5.46	212<br>5.47	261<br>6.44	265<br>6.48	268<br>6.51	269<br>6.52	292<br>7.39	295<br>7.42	296<br>7.43	
human melanopsin	M	T	Q	**F**	Y	G	A	G	I	A	E	S	**S**	**W**	**L**	C	V	F	F	W	Y	**S**	A	A	K	
bovine melanopsin	M	T	Q	**F**	Y	G	A	G	I	A	E	S	**S**	**W**	**L**	F	V	F	F	W	Y	**S**	A	A	K	
mouse melanopsin	M	T	Q	**F**	Y	G	A	G	I	A	E	S	**S**	**W**	**L**	F	V	F	F	W	Y	**S**	A	A	K	
anole Opn4	M	T	Q	**F**	Y	G	A	G	I	S	E	S	**S**	**W**	**L**	F	V	F	Y	W	Y	**S**	A	A	K	*Opn4m*
chicken Opn4-2	M	T	Q	**F**	Y	G	A	G	I	S	E	S	**S**	**W**	**L**	F	V	F	Y	W	Y	**S**	A	A	K	
zebrafish Opn4a	M	T	Q	**F**	Y	G	A	G	I	S	E	S	T	**W**	**L**	F	V	F	Y	W	Y	**S**	A	A	K	
zebrafish Opn4b	M	T	Q	**F**	Y	G	A	G	I	S	E	S	**S**	**W**	**L**	F	V	F	Y	W	Y	**S**	A	A	K	
lamprey Opn4	M	T	Q	**F**	Y	G	A	G	I	S	E	S	T	**W**	**L**	F	V	F	Y	W	Y	**S**	A	A	K	
Xenopus Opn4x	M	T	Q	C	Y	G	A	G	I	S	E	S	T	**W**	**L**	C	V	F	F	W	Y	A	A	A	K	
chicken Opn4-1	M	S	Q	C	Y	G	A	G	I	S	E	S	T	**W**	**L**	C	V	F	Y	W	Y	A	A	A	K	*Opn4x*
zebrafish Opn4xa	M	T	Q	**F**	Y	G	A	G	I	S	E	S	T	**W**	**L**	C	V	F	F	W	Y	A	A	A	K	
zebrafish Opn4xb	M	T	Q	**F**	Y	G	A	G	I	S	E	S	T	**W**	**L**	C	V	F	Y	W	Y	A	A	A	K	
belcheri melanopsin	M	T	N	**F**	Y	G	G	G	C	S	E	S	T	F	I	F	M	Y	F	W	Y	A	A	A	K	*invertebrate melanopsin*
lanceolatum melanopsin	M	T	N	**F**	Y	G	G	G	C	S	E	S	**S**	F	I	F	M	Y	F	W	Y	A	A	A	K	
jumping spider rhodopsin-1	M	F	M	M	Y	G	S	G	S	S	E	S	T	I	Y	A	V	Y	W	W	Y	L	A	A	K	*Gq-coupled visual opsin*
bovine rhodopsin	M	G	G	T	E	A	T	G	E	A	E	S	G	I	M	F	H	F	F	W	Y	A	A	A	K	*outgroup*

Supplemental Fig. S2 Obayashi et al.

```
                                                                                                TM1
human_melanopsin     -----------------MNPPSGPRVPPSPTQEPSCMATPAPPSWWDSSQSSISSLGRLPSISPTAPGTWAAAWVPLPTVDVPDHAH
mouse_melanopsin     -----------------MDSPSGPRVLSSLTQDPSFTTSPALQGIWNGTQN-VSVRAQLLSVSPTTSAHQAAAWVPFPTVDVPDHAH
Xenopus_Opn4x        ------------------------------------------------------------MDLGKTVEYGTHRQDAIAQIDVPDQVL
chicken_Opn4-1       ------------------------------------------------------------MDLNMGTQP-----HSVTKSEIPDHVL
lanceolatum_mela     ----------------------------------------------------MEVTPTPGCTGNASVCNGTDSGGGGVVWDIPPLAH
belcheri_mela        MTEIPSFQPPINATEVEEENAVFPTALTEWFSEVGNQVGEVALKLLSGEGDGMEVTPTPGCTGNGSVCNGTDSG--GVVWDIPPLAH
jumping_spider_rh1   -------------------------------------MLPHAAKMAARVAGDHDGRNISIVDLLPEDMLPMIHEHWYKFPPMETSMH
Bovine_rhodopsin     --------------------------------------------------MNGTEGPNFYVPFSNKTGVVRSPFEAPQYYLAEPWQF

                                                                            94^2.61
                               TM1                                 TM2         ▼                          TM3
human_melanopsin     YTLGTVILLVGLTGMLGNLTVIYTFCRSRSLRTPANMFIINLAVSDFLMSFTQAPVFFTSSLYKQWLFGETGCEFYAFCGALFGISS
mouse_melanopsin     YTLGTVILLVGLTGMLGNLTVIYTFCRNRGLRTPANMFIINLAVSDFLMSVTQAPVFFASSLYKKWLFGETGCEFYAFCGAVFGITS
Xenopus_Opn4x        YTIGSFILIIGSVGIIGNMLVLYAFYRNKKLRTAPNYFIINLAISDFLMSATQAPVCFLSSLHREWILGDIGCNVYAFCGALFGITS
chicken_Opn4-1       YTVGTCVLVIGSIGIIGNLLVLYAFYSNKKLRTPQNFFIMNLAVSDFLMSASQAPICFINSLHREWILGDIGCDLYAFCGALFGITS
lanceolatum_mela     YIVGTAVFCVGCCGMFGNAVVVYSFIKAKGLRTPANFFIVNLALSDLLMNLTNMPLFAVNSAFQRWLLSDFACELYGFAGGLFGCLS
belcheri_mela        YIVGTAVFCIGCCGMFGNAVVVYSFIKSKGLRTPANFFIINLALSDFLMNLTNMPIFAVNSAFQRWLLSDFACELYGFAGGLFGCLS
jumping_spider_rh1   YILGMLIIVIGIISVSGNGVVMYLMMTVKNLRTPGNFLVLNLALSDFGMLFFMMPTMSINCFAETWVIGPFMCELYGMIGSLFGSAS
Bovine_rhodopsin     SMLAAYMFLLIMLGFPINFLTLYVTVQHKKLRTPLNYILLNLAVADLFMVFGGFTTTLYTSLHGYFVFGPTGCNLEGFFATLGGEIA
                                       ▲                     ▲
                                     55^1.50               83^2.50

                                                                           188^ECL2 189^ECL2        207^5.42
                     TM3                        TM4                  ECL2          ▲ ▲                  ▼ TM5
human_melanopsin     MITLTAIALDRYLVITRPLATFGVASKRRAAFVLLGVWLYALAWSLPPFFGWSAYVPEGLLTSCSWDYMSFTPAVR--AYTMLLCCF
mouse_melanopsin     MITLTAIAMDRYLVITRPLATIGRGSKRRTALVLLGVWLYALAWSLPPFFGWSAYVPEGLLTSCSWDYMTFTPQVR--AYTMLLFCF
Xenopus_Opn4x        MMTLLAISINRYIVITKPLQSIQWSSKKRTSQIIVLVWMYSLMWSLAPLLGWSSYVPEGLRISCTWDYVTSTMSNR--SYTMMLCCC
chicken_Opn4-1       MMTLLAISVDRYLVITKPLRSIQWTSKKRTIQIIAAVWLYSLGWSVAPLLGWSSYVPEGLMISCTWDYVTYSPANR--SYTMILCCC
lanceolatum_mela     INTLMAISMDRYLVITKPFLVMRIVTKQRVMFAILLLWVWSLVWSLPPLFGWGAYVSEGFGTSCSFDYMTPKLSFH--IFTYFIFFT
belcheri_mela        INTLMAISMDRYLVITKPFLVMRIVTKQRVMFAILLLWIWSLVWALPPLFGWSAYVSEGFGTSCTFDYMTPKLSYH--IFTYIIFFT
jumping_spider_rh1   IWSLVMITLDRYNVIVKGMAGKP-LTKVGALLRMLFVWIWSLGWTIAPMYGWSRYVPEGSMTSCTIDYIDTAINPM--SYLIAYAIF
Bovine_rhodopsin     LWSLVVLAIERYVVVCKPMSNFR-FGENHAIMGVAFTWVMALACAAPPLVGWSRYIPEGMQCSCGIDYYTPHEETNNESFVIYMFVV
                               ▲                         ▲
                            135^3.50                  161^4.50

                                                                                             269^6.52
                      TM5                                                      TM6              ▼
human_melanopsin     VFFLPLLIIIYCYIFIFRAIR----ETGRALQTFGACKGNGESLWQRQRLQSECKMAKIMLLVILLFVLSWAPYSAVALVAFAGYAH
mouse_melanopsin     VFFLPLLIIIFCYIFIFRAIR----ETGRACEGCGESPLRQRRQWQR--LQSEWKMAKVALIVILLFVLSWAPYSTVALVAFAGYSH
Xenopus_Opn4x        VFFIPLIVISHCYLFMFLAIR----STGRNVQKLG---SYGRQSFLSQSMKNEWKMAKIAFVIIIVFVLSWSPYACVTLIAWAGHGK
chicken_Opn4-1       VFFIPLIIILHCYLFMFLAIR----STGRDVQKLG---SCSRKSFLSQSMKNEWKLAKIAFVVIIVYVLSWSPYACVTLIAWAGRGN
lanceolatum_mela     MYFIPLGVIIYCYYNIFATVKSGDKQFGKAVKEMAHEDVKNKAQQERQ-RKNEIKTAKISFIVITLFISAWTPYAVVAALGTLGYQH
belcheri_mela        MYFIPGGVMIYCYYNIFATVKSGDKQFGKAVKEMAHEDVKNKAQQERQ-RKNEIKTAKIAFIVISLFMSAWTPYAVVSALGTLGYQD
jumping_spider_rh1   VYFVPLFIIIYCYAFIVMQVAAHEKSLREQAKKMN--IKSLRSNEDNKKASAEFRLAKVAFMTICCWFMAWTPYLTLSFLGIFSDRT
Bovine_rhodopsin     HFIIPLIVIFFCYGQLVFTVK----------------EAAAQQQESATTQKAEKEVTRMVIIMVIAFLICWLPYAGVAFYIFTHQGS
                         ▲                                                                  ▲
                      215^5.50                                                           267^6.50

                                 TM7
human_melanopsin     VLTPYMSSVPAVIAKASAIHNPIIYAITHPKYRVAIAQHLPCLGVLLGVSRRHSRPYPS
mouse_melanopsin     ILTPYMSSVPAVIAKASAIHNPIIYAITHPKYRVAIAQHLPCLGVLLGVSGQRSHPSLS
Xenopus_Opn4x        SLTPYSKTVPAVIAKASAIHNPIIYAITHPKYRVAIAQHLPCLGVLLGVSGQRSHPSLS
chicken_Opn4-1       TLTPYSKSVPAVIAKASAIYNPIIYGIIHPKYRETIHKTVPCLRFLIREPKKDIF
lanceolatum_mela     LVTPYLQSIPAVIAKASAIYNPIIYAIIHPRYRKTIHNAVPCLRFLIRISKNDLL
belcheri_mela        LVTPYLQSIPAMFAKSSAVYNPIVYAITHPKFRAAVKKHIPCLSGCLPADEEETKTKTRGA
jumping_spider_rh1   WLTPMTSVWGAIFAKASACYNPIVYGISHPKYRAALHDKFPCLKCGSDSPKGDSASTVAESEKAGE
Bovine_rhodopsin     DFGPIFMTIPAFFAKTSAVYNPVIYIMMNKQFRNCMVTTLCCGKNPLGDDEASTTVSKTETSQVAPA
                                         ▲
                                      303^7.50
```

Supplemental Fig. S3 Obayashi et al.

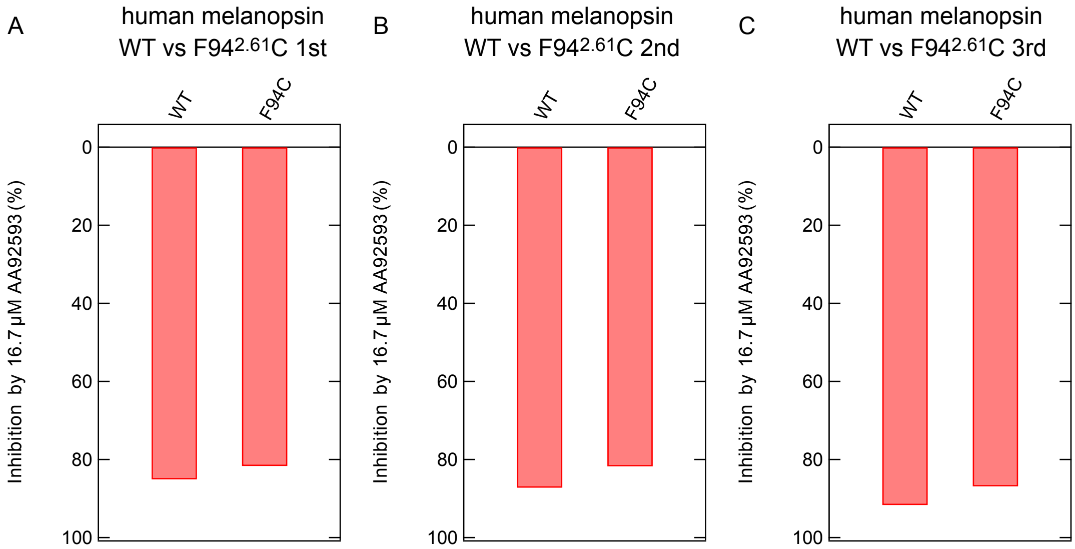

Supplemental Fig. S4 Obayashi et al.

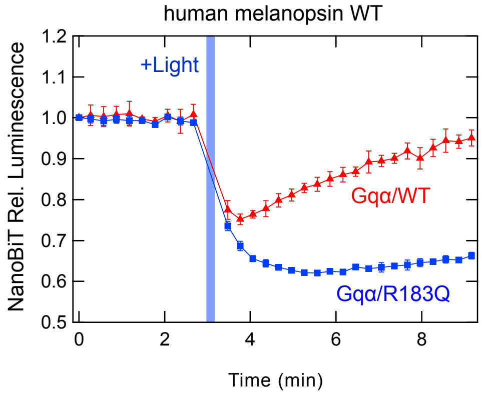

Supplemental Fig. S5 Obayashi et al.

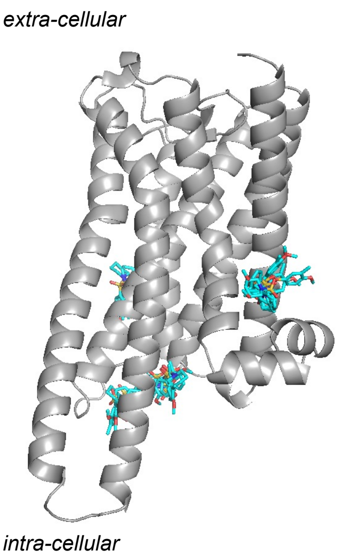

Supplemental Fig. S6 Obayashi et al.

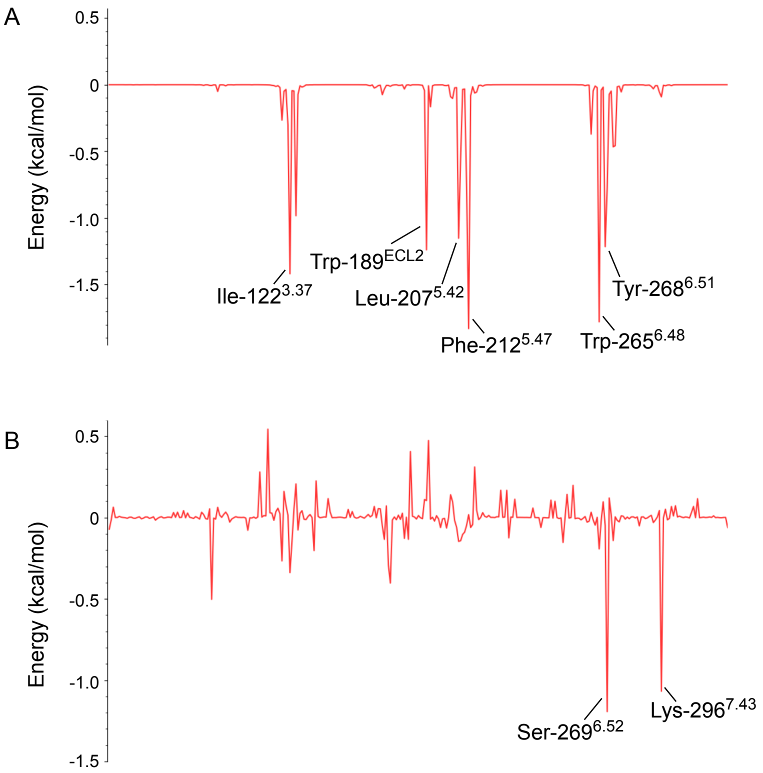

Supplemental Fig. S7 Obayashi et al.

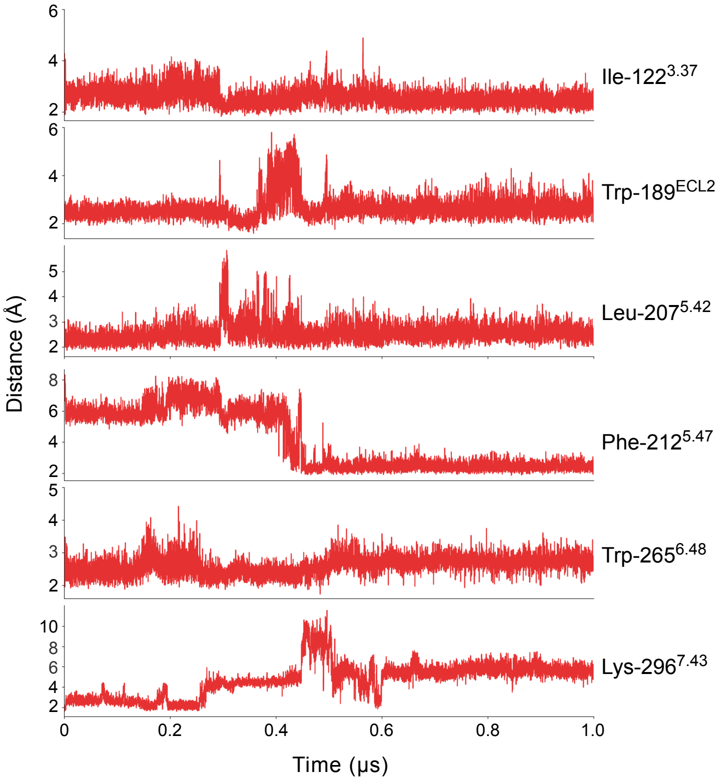

Supplemental Fig. S8 Obayashi et al.

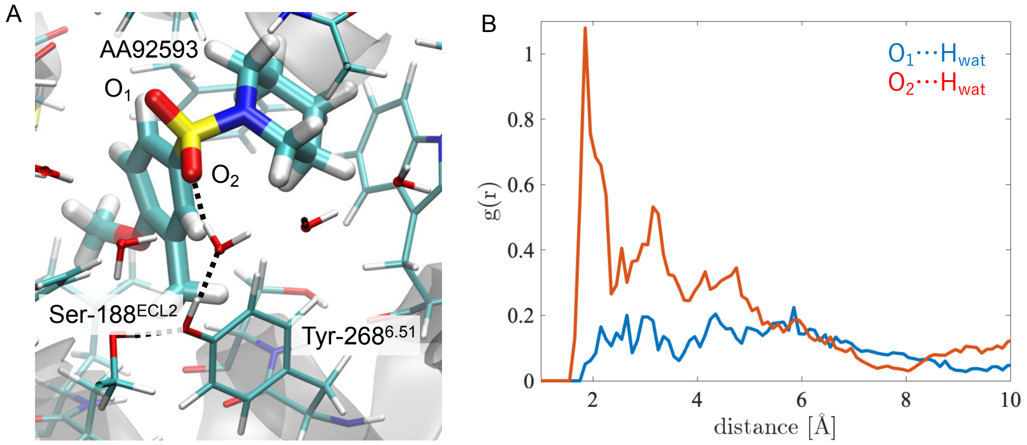

Supplemental Fig. S9 Obayashi et al.

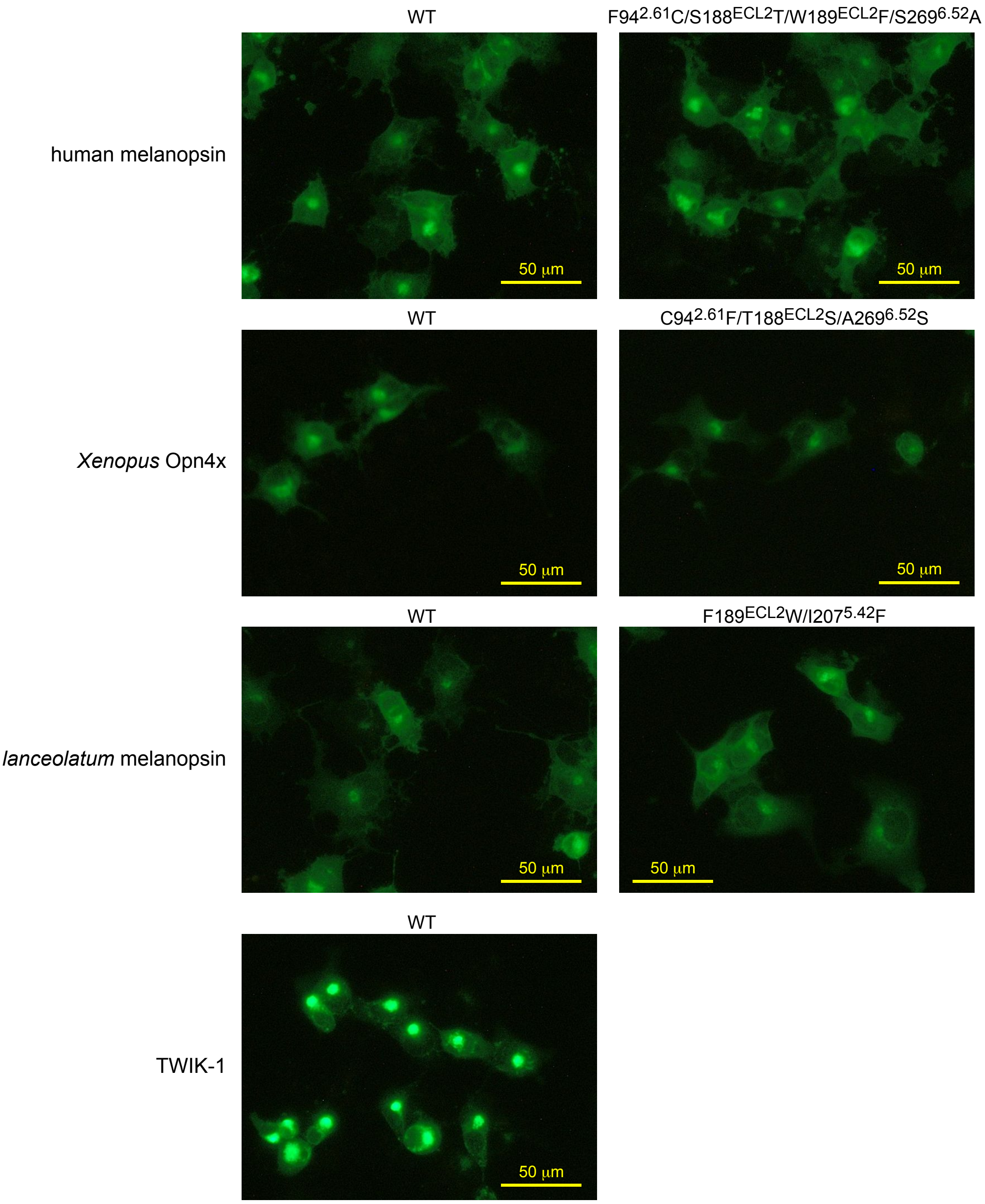

Supplemental Fig. S10 Obayashi et al.

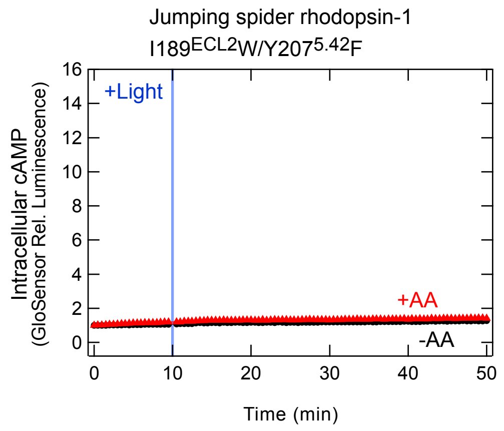

Supplemental Fig. S11 Obayashi et al.
